# The association between breastmilk oligosaccharides and faecal microbiota in healthy breastfed infants at two, six, and twelve weeks of age

**DOI:** 10.1038/s41598-020-61024-z

**Published:** 2020-03-06

**Authors:** Klaudyna Borewicz, Fangjie Gu, Edoardo Saccenti, Christine Hechler, Roseriet Beijers, Carolina de Weerth, Sander S. van Leeuwen, Henk A. Schols, Hauke Smidt

**Affiliations:** 1 Laboratory of Microbiology, Wageningen University & Research, Stippeneng 4, 6708 WE Wageningen, The Netherlands; 2 Laboratory of Food Chemistry, Wageningen University & Research, Bornse Weilanden 9, 6708 WG Wageningen, The Netherlands; 3 Laboratory of Systems and Synthetic Biology, Wageningen University & Research, Stippeneng 4, 6708 WE Wageningen, The Netherlands; 4 Department of Developmental Psychology, Behavioral Science Institute, Radboud University, 6500 HE Nijmegen, The Netherlands; 5 Department of Cognitive Neuroscience, Donders Institute for Brain, Cognition and Behaviour, Radboud University Medical Center, 6525 HR Nijmegen, The Netherlands; 6 Microbial Physiology, Groningen Biomolecular Sciences and Biotechnology Institute (GBB), University of Groningen, Nijenborgh 7, 9747 AG Groningen, The Netherlands; 7 Present Address: Sector Human Nutrition & Health, Department of Laboratory Medicine, University Medical Center Groningen, Hanzeplein 1, 9713 GZ Groningen, The Netherlands

**Keywords:** Microbial ecology, Microbiome, Nutrition, Paediatric research

## Abstract

Several factors affect gut microbiota development in early life, among which breastfeeding plays a key role. We followed 24 mother-infant pairs to investigate the associations between concentrations of selected human milk oligosaccharides (HMOs) in breastmilk, infant faeces, and the faecal microbiota composition in healthy, breastfed infants at two, six and 12 weeks of age. Lactation duration had a significant effect on breastmilk HMO content, which decreased with time, except for 3-fucosyllactose (3FL) and Lacto-*N*-fucopentaose III (LNFP III). We confirmed that microbiota composition was strongly influenced by infant age and was associated with mode of delivery and breastmilk LNFP III concentration at two weeks, with infant sex, delivery mode, and concentrations of 3′sialyllactose (3′SL) in milk at six weeks, and infant sex and Lacto-*N*-hexaose (LNH) in milk at 12 weeks of age. Correlations between levels of individual breastmilk HMOs and relative abundance of OTUs found in infant faeces, including the most predominant *Bifidobacterium* OTUs, were weak and varied with age. The faecal concentration of HMOs decreased with age and were strongly and negatively correlated with relative abundance of OTUs within genera *Bifidobacterium, Parabacteroides*, *Escherichia-Shigella, Bacteroides, Actinomyces, Veillonella*, Lachnospiraceae *Incertae Sedis*, and Erysipelotrichaceae *Incertae Sedis*, indicating the likely importance of these taxa for HMO metabolism *in vivo*.

## Introduction

Microbial colonisation of the infant gastrointestinal (GI) tract begins before or at birth, and in healthy, breastfed infants it progresses towards a microbial community that is dominated by bifidobacteria and is metabolically adapted to thrive on human milk^1,2^. Many host specific and environmental factors have been identified to play a role in the development of human GI tract microbiota^3^, and understanding of the impact of these factors and their associated health outcomes has been a growing area of research during recent years^4,5^. Breastfeeding is essential for optimal colonization and maturation of the infant GI microbiota; breastmilk not only provides an important medium for the transfer of microbes between the mother and her infant, but it also contains high concentrations of prebiotic human milk oligosaccharides (HMOs) which further facilitate microbial colonisation^6^. High concentrations of HMOs in breastmilk are believed to be the main force shaping the bifidobacteria dominated GI ecosystem in breastfed infants, although up to date only few *in vivo* studies have been able to demonstrate this^2,7^. The type and amount of the secreted HMOs in breastmilk are genetically predetermined, influenced by maternal and other factors, and are highly variable among mothers and across lactation stages^8–12^. Up to date over 200 different HMO structures have been identified^13^ and this variability in the chemical and structural conformations of HMOs might be biologically relevant. Colonisation and growth of highly specialised taxa and consequently the progression of the microbial succession within the infant gut might be supported by specific HMO types. The specific utilisation of individual HMOs by infant’s colon microbiota measured in the faeces during the first month after birth has been reported by Albrecht *et al*.^14,15^. Microbiota composition during infancy plays an important role in processes that have life-long health consequences, such as educating the immune system, metabolic programming, and facilitating nutrient utilisation^6^. Thus, it is important to gain better understanding of the biological function of the different HMOs in shaping microbiota development and function. Until now, there have been no reports on longitudinal studies investigating the establishment of infant GI microbiota in relation to changes in breastmilk HMO composition, and previous studies focused mainly on *in vitro* fermentation of HMOs by faecal bacterial inoculum, or by faecal isolates^16^. Little is known about how the GI microbial community development affects an infant’s ability to digest different HMOs, and how this ability changes during early infancy. In order to fill these knowledge gaps, we followed a cohort of 24 full term, healthy, breastfed Dutch infants and analysed maternal breastmilk and infant faecal samples collected at two, six and 12 weeks post-delivery. We measured 18 highly abundant HMOs, which account for approximately 86% of the total HMOs in breastmilk^17^, including 13 neutral and five acidic HMOs (Table S1)^18^. We measured the association between the concentrations of these breastmilk HMOs and infant faecal microbial composition through the first three months of life. We also investigated how the microbiota composition correlated with the changes in faecal HMO concentrations, as a proxy for the *in vivo* degradation of the measured HMOs.

## Results

The infants included in this study were delivered vaginally (n = 22) or via C-Section (n = 2). For one of the infants, data on sex, weight and place of delivery were not reported. The remaining infants included 13 boys and ten girls, and their average body weight at birth was 3,536 g and ranged from 3,024 g to 4,140 g. Five infants were born at a clinic, seven at home, nine at a hospital, and for two infants birth started at home but was completed at a hospital. Six infants had a common cold and one had diarrhoea during the study. The actual ages in days ranged within each collection time point; at two weeks time point infant age ranged from seven to 20 d (*M* = 14.28, *SD = *2.06), at six weeks it was 41 d to 44 d (*M* = 41.12, *SD = *1.42), and at 12 weeks it was 80 d to 93 d (*M* = 84.42, *SD = *2.48).

The Illumina HiSeq sequencing resulted in a total number of 8,550,719 (range: 3,383-421,482 per sample, *M* = 118,760, *SD = *86,261, *SE* = 10,166) sequencing reads that passed the quality filtering (Fig. S1). A total number of 411 Operational Taxonomic Units (OTUs) were identified of which 83 OTUs were found in more than 5%, or at least four samples (Table S2).

### The development of infant faecal microbiota

**Figure 1:**
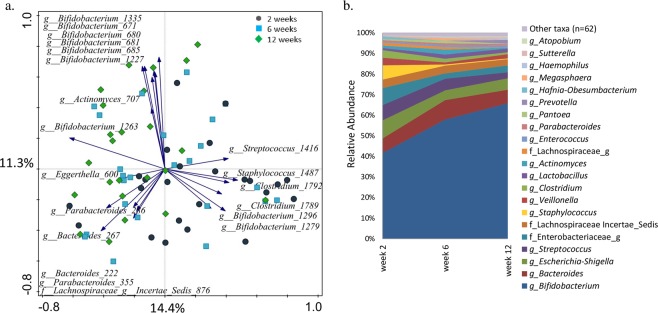
Infant faecal microbiota composition. (**a**) PCA showing spatial distribution of samples from 24 infants based on their faecal microbiota composition at two, six and 12 weeks of age and the twenty best fitting OTUs; (**b**) Average relative abundance of genus level taxa in the 24 infants at different time points. In case the taxonomic assignment could not be made at genus level, the lowest classifiable taxonomy assignment is used, and the unidentified genus is indicated with “_g”.

Alpha diversity analyses showed no statistically significant differences in richness and diversity in faecal microbiota of infants at different time points when tested with Kruskal-Wallis and Wilcoxon tests (Fig. S2). The Principal Component Analysis (PCA) showed that composition of faecal microbiota of individual infants was diverse, but despite the high inter-individual variation, the observed changes in microbial profiles were directional and progressed with time towards bifidobacteria-dominated communities (Fig. 1). Redundancy analysis (RDA) was performed on the OTU level faecal microbiota data, and together all explanatory variables could explain 56.58% of variation in infant microbiota, with the statistically significant effect (False Discovery Rate (FDR) < 0.05) of infant age (4.3% variation explained), sex (6.1%), place (8.2%) and mode of delivery (3.5%), secretor status (1.7%), and the breastmilk HMO concentrations of 6′-sialyllactose (6′SL) (5.1%), 3′-sialyllactose (3′SL) (3.2%), 2′-fucosyllactose (2′FL) (2.9%), Lacto-*N*-difucohexaose II (LNDFH II) (2.6%), and 3FL (2%). RDA was repeated separately at each time point, and showed that infant faecal microbiota composition was associated (FDR > 0.05; p < 0.05) at two weeks of age with mode of delivery (6.7% variation explained) and breastmilk LNFP III levels (8.1%), at six weeks with infant sex (6.9%), mode of delivery (6.3%) and breastmilk 3′SL levels (7.3%), and at 12 weeks with sex (7.2%) and breastmilk LNH levels (6.6%).

### Milk HMO content and changes during lactation

**Figure 2:**
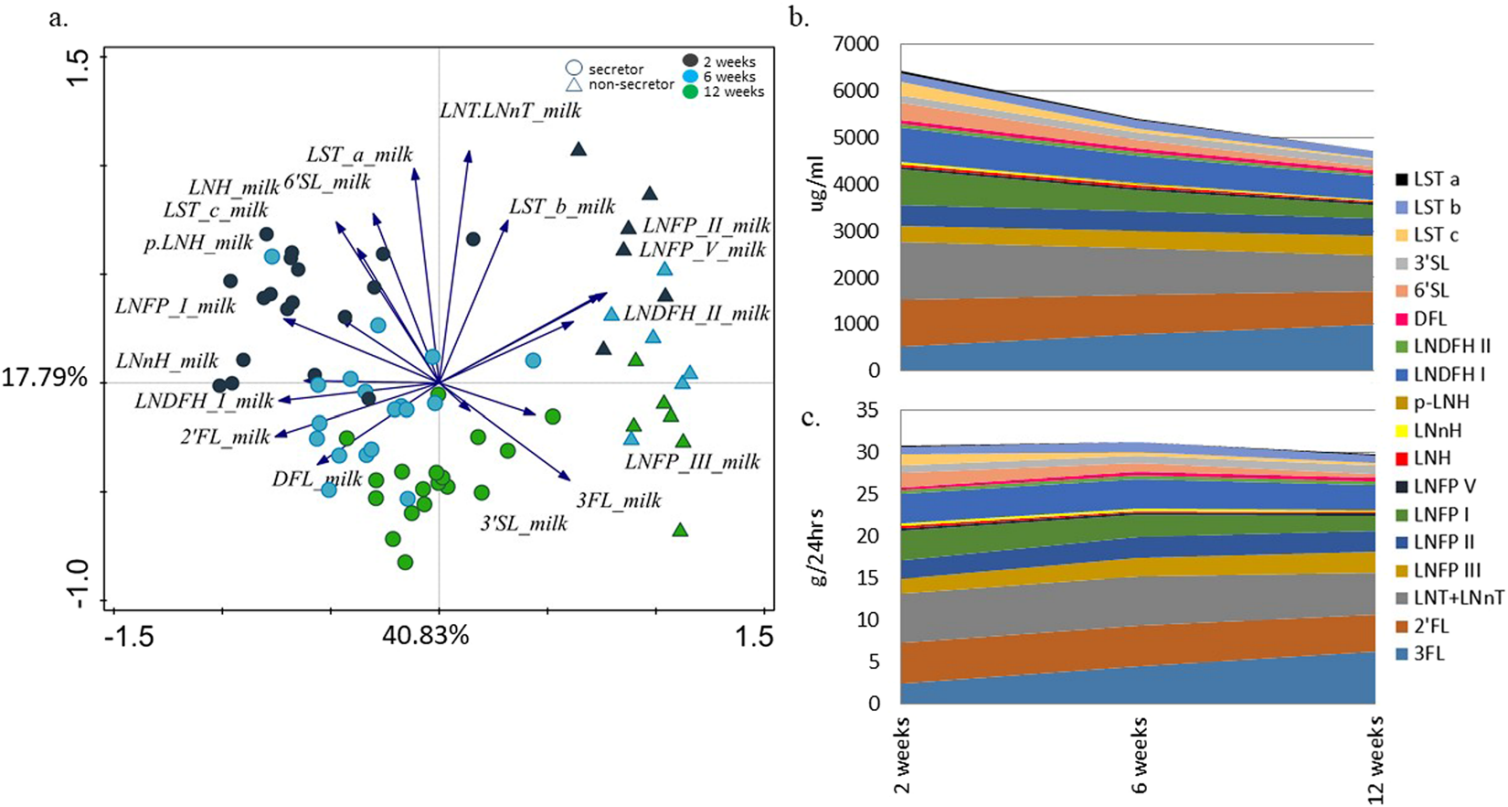
Breastmilk HMO profiling. (**a**) PCA showing separate clustering of breastmilk samples of 24 mothers at two, six and 12 weeks of lactation, also indicating mother’s secretor status. The 3FL, LNFP III, and 3SL (3SL between week six and 12 only) are positively associated with duration of lactation; (**b**) Average concentration of different HMOs found in the breastmilk samples from the 24 mothers included in the study at two, six and 12 weeks of lactation; (**c**) Estimated average daily intake of each HMO in the 24 infants at two, six and 12 weeks of age.

HMO profiles of each breastmilk sample were analysed (Table S3a), and samples from six mothers revealed consistent lack of 2′FL and low levels of Lacto-N-fucopentaose I (LNFP I), Lacto-N-difucohexaose I (LNDFH I) and Difucosyllactose (DFL) (non-secretors). PCA of milk HMOs showed a clear separation of samples in relation to collection time point postpartum and secretor status (Fig. 2a). This finding was confirmed with RDA, which showed that collection time point and secretor status had a significant effect on the types and levels of the HMOs present, explaining respectively 16.7% and 2.5% of variation in the data. When analysed separately for each time point, neither the mode of delivery nor maternal stress measured via saliva cortisol, were significantly associated with the HMO levels in mothers’ milk (data not shown). The average concentration (µg/ml) of the HMOs measured in the milk decreased with time of lactation, except for the concentrations of 3FL and LNFP III, which were secreted at higher amounts at later time points (Fig. 2b, Table S3a). After adjusting the HMO concentrations for the literature-based averages^19^ of the amounts of milk consumed (480 g, 580 g and 630 g at weeks two, six and 12, respectively), the estimated average HMO intake remained stable in the first three months of life (Fig. 2c).

### Correlation between milk HMOs and infant faecal microbiota

**Figure 3:**
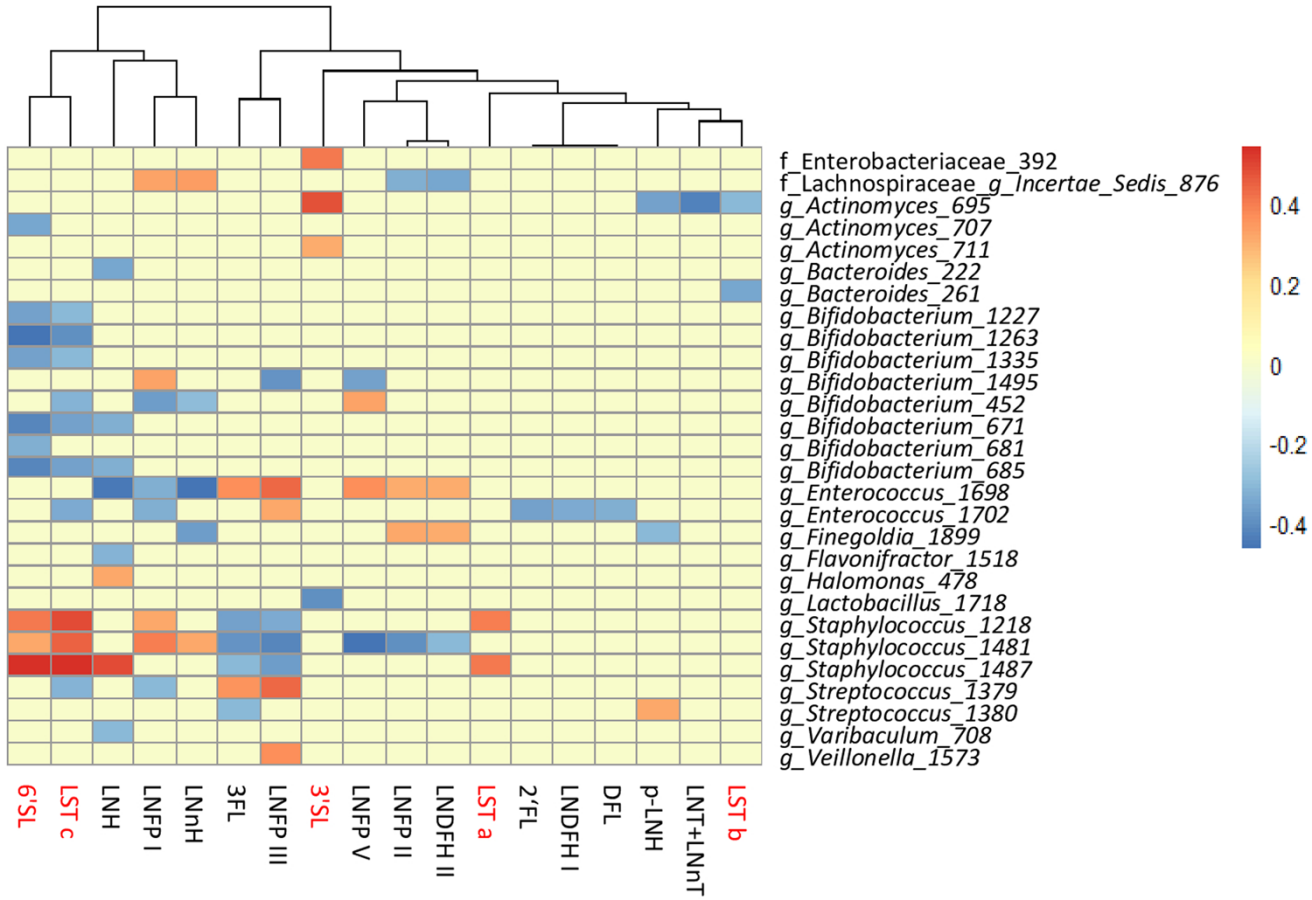
Statistically significant (p < 0.05) Spearman correlations (correlation threshold ±0.3) between estimated daily intake of different milk HMOs and faecal microbiota composition at OTU level of 24 infants across the study duration. Positive associations are indicated in red, negative in blue, and correlations that did not pass significance or correlation threshold are marked in yellow. The names of acidic HMOs are highlighted in red.

The estimated daily intake (g/day) of each HMO in each infant, and the relative abundance of OTUs for the corresponding faecal samples were correlated to examine the potential link between the levels of ingested HMOs and the abundance of different microbial taxa in infant faeces (Fig. 3). The strongest positive correlations were detected between few of the *Staphylococcus* OTUs and milk levels of 6′SL, Sialyl-lacto-N-tetraose c (LST c), Sialyl-lacto-N-tetraose a (LST a), LNH, Lacto-N-neohexaose (LNnH), and LNFP I. *Streptococcus* OTUs were positively correlated with 3FL, LNFP III, and Para-lacto-N-hexaose (pLNH). Positive correlations were also found between OTUs within the genus *Actinomyces* and 3′SL, and *Enterococcus* OTUs and 3FL, Lacto-N-fucopentaose II (LNFP II), LNFP III, Lacto-N-fucopentaose V (LNFP V) and LNDFH II. No significant positive correlations were found between the most predominant *Bifidobacterium* OTU 1263 and any of the milk HMOs. Spearman correlation analysis at each separate time point (Fig. S3) showed that at two weeks of age three bifidobacterial OTUs were positively associated with LNH, pLNH and LNFP I, and the main *Bifidobacterium* OTU 1263 was negatively associated with LNFP II and LNFP III (Fig. S3a). At six weeks three different low abundance bifidobacterial OTUs were positively associated with LNFP V, LNnH, 6′SL, Sialyl-lacto-N-tetraose b (LST b) and LST c (Fig. S3b), and at 12 weeks LNnH, LNFP II, and LNDFH II showed a low positive correlation with two low abundance bifidobacterial OTUs. Overall the associations did not exceed ±0.6, there was no consistency in the type (positive or negative), or strength (passing threshold of ±0.3, p < 0.05) of associations between specific OTU-HMO pairs when data from all time points was combined, or when individual time points were analysed separately.

### Correlation between infant faecal microbiota and HMOs excreted in infant faeces

**Figure 4:**
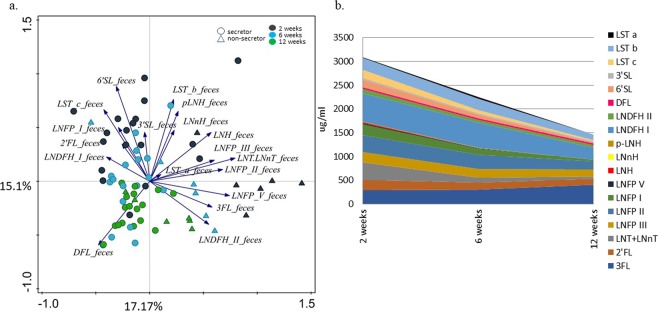
Faecal HMO profiling. (**a**) PCA showing spatial distribution of faecal samples of 24 infants at two, six and 12 weeks of age based on the concentrations of residual HMOs detected in infant faeces, and indicating mother’s secretor status; (**b**) Average concentration of different HMOs (µg/ml of faeces) found in infant faeces decreases with infant age, except for 3FL, which for most infants increased with age.

**Figure 5:**
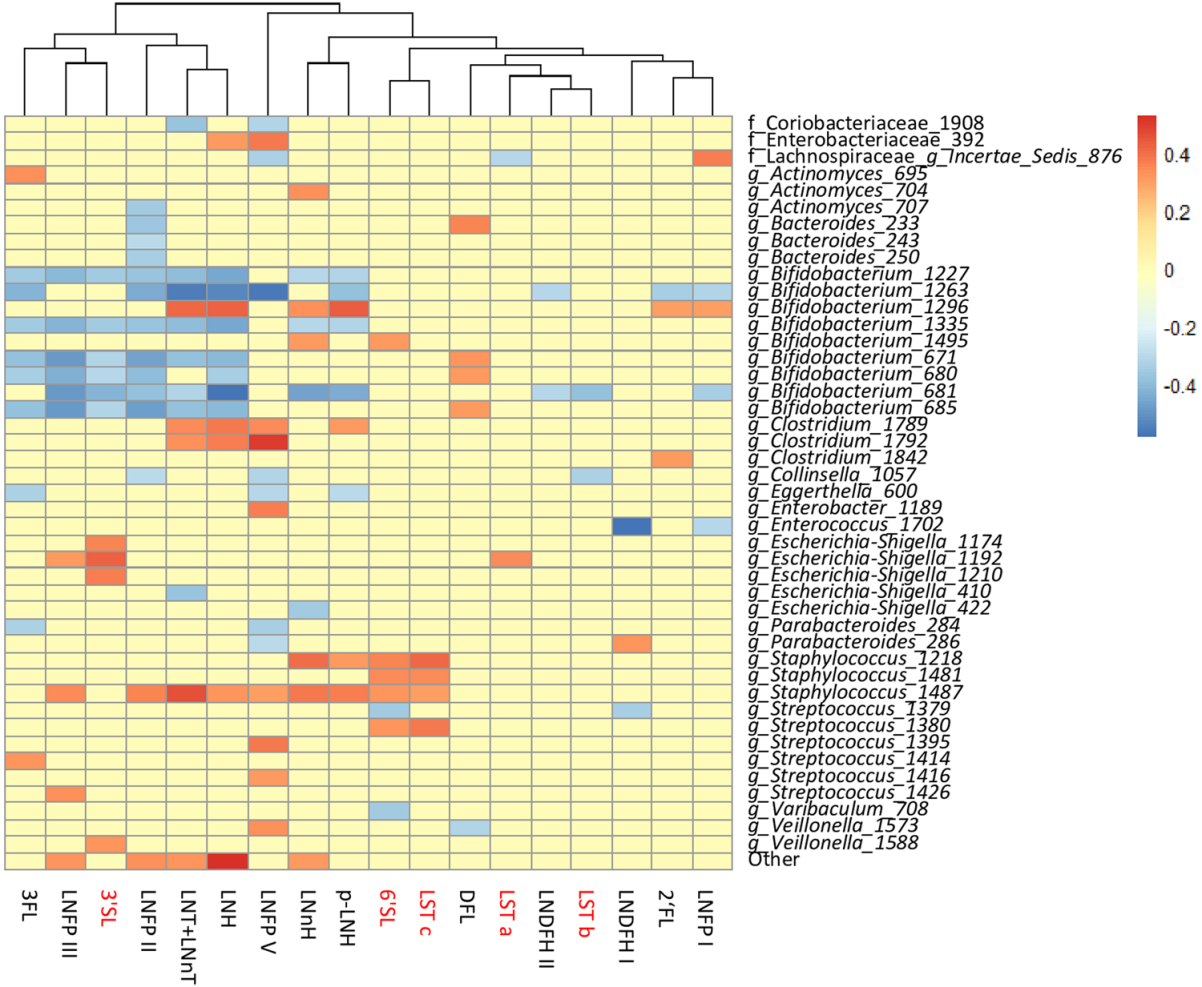
Statistically significant (p < 0.05) Spearman correlations (correlation threshold ± 0.3) between HMOs detected in infant faeces and faecal microbiota composition at OTU level of 24 infants across the study duration. Positive associations are indicated in red, negative in blue, and correlations that did not pass significance or correlation threshold are marked in yellow. The names of acidic HMOs are highlighted in red.

Undigested milk HMOs were secreted in infant faeces and their concentrations varied between infants and across study time points (Table S3b). Our hypothesis was that aside of being directly dependent on the amounts of the HMOs ingested with the milk, the faecal HMO concentrations could serve as an indicator of the utilisation of different HMOs by infant GI tract microbiota. PCA (Fig. 4a) showed that samples clustered by infant age, and to lesser extent also by the maternal secretor status. RDA confirmed these findings showing a significant (FDR < 0.05) effect of infant age (7.5% explained variation) and maternal secretor status (7.3%) on the HMO concentrations in infant faeces (Figure not shown). The concentrations of all HMOs in faeces decreased with infant age, with the exception of 3FL (Fig. 4b). Spearman correlation between the faecal HMO concentrations and faecal microbiota showed that the majority of statistically significant negative associations were between bifidobacterial OTUs and thirteen different HMOs (Fig. 5). The main *Bifidobacterium* OTU 1263 was negatively correlated with nine different HMOs, of which LNH, LNFP V, and the LNT/LNnT group showed strongest correlations, suggesting an important role of *Bifidobacterium* OTU 1263 in consumption of these HMOs in the infant GI tract. Positive associations were also observed between various OTUs of *Streptococcus, Staphylococcus, Clostridium* and *Escherichia-Shigella* and thirteen different HMOs. Spearman correlation analysis at each separate time point showed that at two weeks of age the strongest negative correlations were found between various HMOs and highly abundant *Bifidobacterium* OTU 1263 and 681 (Fig. S4a). At week six, an additional six bifidobacterial OTUs showed negative correlations with pLNH, LNH, LNFP II and III. At both time points *Bifidobacterium* OTU 1263 was the only one correlating negatively with 3FL, possibly highlighting the unique link between this HMO and the major *Bifidobacterium* OTU during the initial stages of the development of GI tract microbiota (Fig. S4b). At 12 weeks of age, the HMO concentrations in faeces were very low or no longer within the detectable range, leading to overall less clear correlation patterns between bifidobacteria and faecal HMOs. Still, at 12 weeks of age *Bifidobacterium* OTU 1263 correlated strongly with 2′FL, LNDFH II and LNFP V (Fig. S4c).

**Figure 6:**
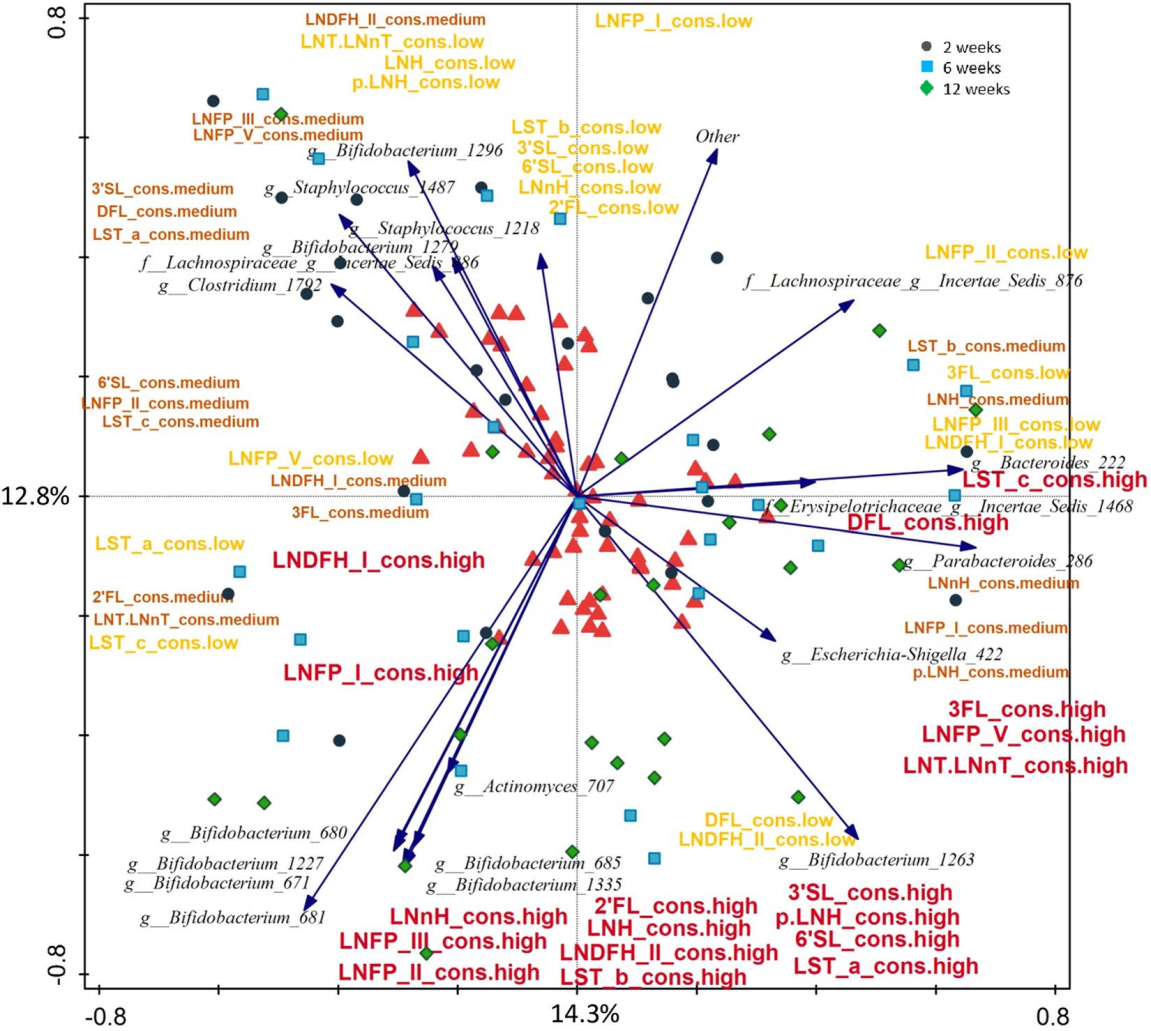
RDA showing spatial distribution of faecal samples of 24 infants at two, six and 12 weeks of age based on their OTU composition and using the estimated level of consumption (low, medium and high) for each HMO as explanatory variables. The red triangles represent centroids of each consumption group (high, medium and low) for each of the HMOs. For better image clarity only top twenty best fitted microbial taxa are displayed.

**Figure 7:**
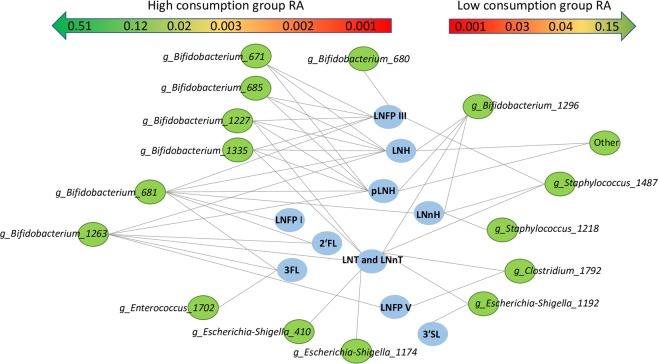
Differentially abundant OTUs in faecal samples of infants classified into high or low HMO consumption categories for each HMO type (Kruskal-Wallis; FDR < 0.05). OTUs enriched in infants classified as high consumers are displayed on the left half of the figure, and OTUs enriched in infants classified as low consumers are displayed on the right half of the figure. The approximate RA of the OTUs is indicated by the gradient arrows on top of the figure.

In order to account for the initial availability of the different HMOs in the milk, we calculated ratios between each faecal and milk HMO, for each mother-infant pair at each time point. The resulting ratios were then used to estimate the consumption level for each HMO as either “high”, “medium” or “low” based on tertiles. RDA showed that high consumption was associated with older infant age and higher levels of bifidobacteria. Low consumption of pLNH, 2′FL, LNH, LNnH, LNTandLNnT, LNFP I and V, 3′SL and LNDFH I, medium consumption of LNDFH II, LNFP II and 6′SL, and high consumption of pLNH, LNH, LNnH, LNFP I and V, LST a, LNTandLNnT, and 3′SL were significantly associated with the microbial composition (FDR < 0.05; Fig. 6). Overall, high consumption was detected in association with various bifidobacterial OTUs including the most predominant *Bifidobacterium* OTU 1263, and several OTUs within genera *Parabacteroides*, *Escherichia-Shigella, Bacteroides, Actinomyces, Veillonella*, and Erysipelotrichaceae *Incertae Sedis* (Figs. 5 and 6). The result was confirmed when relative abundance of OTUs between infants assigned into a low and high consumption groups for each HMO were compared (Table S4) showing significantly higher relative abundance of various bifidobacterial OTUs, and in most cases the most predominant OTU *Bifidobacterium* 1263 in the high consumption group (Fig. 7). Interestingly, high consumption of 6′SL, DFL, LST a, and LST c was not significantly linked with the presence of bifidobacteria, but instead was linked with other bacterial groups (p < 0.05; Table S4).

## Discussion

In the cohort described in this study we observed directional changes in both HMO concentrations in breast milk and in infant faces, and in the composition of infant faecal microbiota. HMO concentrations differed between individuals and depended on lactation time and infant age. Earlier studies showed that faecal microbiota structure is highly dynamic during infancy and is associated with multiple factors, including diet^20–23^. The variation in the faecal microbiota of infants in our study could be explained by infant age, sex, place and mode of delivery and certain milk HMOs, namely 6′SL, 2′FL, 3FL, 3′SL, LNDFH II (p < 0.05). The effect of factors varied with age. Mode of delivery and breastmilk LNFP III concentrations showed a significant association with faecal microbiota structure at two weeks of age. The important role of the mode of delivery in the initial seeding of the GI tract has been previously reported^24,25^ and has been linked to various health outcomes, both in infancy and beyond^26,27^. At six weeks the significant effect of the mode of delivery could still be detected in our data, and also infant sex and milk 3′SL seemed to play a role. A recent study using animal models showed that sex specific, microbiota-independent differences in immunity may lead to the selection of a sex-specific GI microbiota in adult germ free mice^28^. Sex-related differences in faecal microbiota have been also reported in adults^29^, in pre-term^30^, and term infants^31^, yet the timing and the possible mechanisms that might underlie the sex differentiation of GI microbiota in healthy full term infants remain largely unknown. As male infants tend to have a higher daily milk intake, it is possible that they receive higher doses of microbial and HMO components from the mother’s milk^32^. At 12 weeks of age, infant sex and LNH were significantly associated with microbiota, however, the significant association between microbiota composition and mode of delivery was no longer present. With the accumulating evidence linking mode of delivery with various health effects later in life^26,27,33^, it is likely that the microbiota related programming of the host happens soon after birth, or during specific “windows of opportunity”, thus, even when microbiota recovers to its normal state, the long term health effects of such disturbance might persist throughout life^33^.

One of the crucial factors shaping the development of GI microbial community during infancy is the type of feeding that an infant receives, with formula- and breastfed infants showing very different microbial profiles^34–38^. Human milk is a source of prebiotic HMOs which support microbial colonisation in the infant GI tract^7,16,39^. The HMO content in breastmilk is variable and influenced by maternal, environmental and infant feeding practices^12^. The HMO content is highest in colostrum, and the concentrations of HMOs decrease in mature milk^8–10^. Our data agrees with these findings showing that concentrations of the HMOs measured per mL of milk varied between mothers, and decreased between two and 12 weeks of lactation, except for 3FL and LNFP III, which increased in concentration as lactation progressed. Earlier studies showed that the daily intake of milk increases in the first months of life^19^ likely compensating for the decreased HMO concentrations. Considering the daily milk intake, we could conclude that in our study the amounts of ingested HMOs remained relatively stable during the first 12 weeks of life. The exception was in the intake of 3FL and LNFP III which gradually increased in time. Interestingly, this increase in the 3FL and LNFP III intake corresponded with the increase in the faecal concentrations of these two HMOs, suggesting that, on average, their supply likely exceeded the ability of the infant GI tract microbiota to utilise these two HMOs. Furthermore, the role of these structures might go beyond their prebiotic effect, as both 3FL and LNFP III can act as decoys against pathogen binding and infection^40–42^.

One of the signature bacterial groups found in faeces of breastfed infants is the genus *Bifidobacterium*^2^. *In vitro* studies showed that *Bifidobacterium bifidum* JCM1254^39^, *Bifidobacterium longum* subsp. *infantis*, *Bacteroides fragilis* and *Bacteroides vulgatus*^16^ grow well on HMOs as sole carbon source. Thus, we expected to find positive correlations between certain breastmilk HMOs and the bifidobacterial OTUs. We did not measure the absolute abundance of bacteria in infant faeces and are aware of the limitation of using relative abundance data. However, culture-based studies showed that the total faecal bacterial load, as well as *Bifidobacterium* counts tend to increase in the first month of life^35^. Thus, the observed increase in the number of sequencing reads and the relative abundance of bifidobacteria in our data likely reflect the actual increase in the abundance of this group (Fig. S1). We hypothesized that quantity of HMOs might selectively promote growth of either the primary or secondary HMO degraders, leading to increase in their abundance within the microbial community. However, when using data from the three time points combined, we saw an opposite trend - as the predicted daily intake of most HMOs was stable in time, the relative abundance of bifidobacteria was increasing. The analyses repeated for individual time points showed few positive correlations and the results varied between time points (Fig. S3a–c). This could be due to the fact that, in addition to individual HMOs promoting growth of selected microbes capable to utilise this carbon source, there may be other mechanisms controlling the microbial community structure. For example, presence of other breastmilk components, such as lysozyme, secretory IgA and other endogenous factors can suppress growth of certain members of the community and thus, indirectly allow other bacterial species to dominate the infant GI tract ecosystem^35^. Furthermore, the possible effect of HMOs on microbiota may already start in the breastmilk itself^43^. A recent study on human milk investigated the associations between the HMO content and microbiota composition in colostrum and reported strong positive correlations between different HMOs and various microbial groups, including streptococci, staphylococci, enterococci and bifidobacteria, in particular *Bifidobacterium breve* and LNFP III^44^. In the present study, RDA showed a significant association of milk LNFP III with infant faecal microbiota in all time points combined. There was a strong positive association of milk LNFP III with OTUs belonging to the genera *Veillonella*, *Enterococcus* and *Streptococcus* (Fig. S3). Positive associations at all time points for breastmilk 3′SL and unidentified OTUs within family Enterobacteriaceae and *Actinomyces* 695 were also observed. 6′SL was positively associated with clostridia – *Clostridium* 1789 at week two and *Clostridium* 1639 at week 12. Finally, LNFP III was positively associated with *Enterococcus* 1698 at six and 12 weeks. At two and at six weeks Lachnospiraceae *Incertae Sedis* 876 was negatively associated with LNDFH II, *Lactobacillus* 1718 was negatively associated with 3′SL, and *Bifidobacterium* 1295 with LNFP I. Studies on mature breastmilk microbiota and the microbial transfer of microbiota from mother to infant show that breastmilk contains a distinct microbial community and that breastfed infants receive on average nearly 30% of the bacteria from breastmilk and 10% from areolar skin in the first 30 days of life^45^. The study also concluded that the association was lower in older infants, and it was proportional to the frequency of breastfeeding that an infant received^45^. Thus, it is then likely that some of the correlations observed here were due to a combined effect of the HMOs modulating the microbiota of the mother’s milk and the infant GI tract, as well as due to a direct transfer of bacteria during breastfeeding^43^.

Infant GI microbiota plays an important role in energy metabolism via utilising otherwise indigestible HMOs. Our data showed that the average concentrations of faecal HMOs decreased with age, suggesting that microbiota of older infants was more adapted and efficient in degrading these compounds (Fig. 5b). Furthermore, we noted that the increase in efficiency was correlated with the increase in the relative abundance of several bifidobacterial OTUs, but also *Parabacteroides*, *Escherichia-Shigella, Bacteroides, Actinomyces, Veillonella*, and Erysipelotrichaceae *Incertae Sedis* (Fig. 6). High ability to degrade a wide range of HMOs was associated with higher relative abundance of one or more *Bifidobacterium* OTUs confirming the important role of this bacterial group in the HMO metabolism (Fig. 6). Thirteen faecal HMOs negatively correlated with nine different *Bifidobacterium* OTUs, and the highly abundant *Bifidobacterium* OTU 1263 was negatively correlated with nine different HMOs in faeces, especially LNH, LNT and LNnT and LNFP V. Aside of bifidobacteria, members of the genus *Bacteroides* were significantly more abundant in infants who were good degraders of 2′FL, LNFP I, II, V, and pLNH, and *Parabacteroides* in the high degraders of 3FL, LNFP V, LNH, LNT and LNnT, indicating that these microbial groups might have a mutualistic or symbiotic relationship degrading those compounds. In addition, *Halomonas*, *Enterococcus*, *Lactobacillus*, St*aphylococcus, Suterella*, *Varibaculum*, *Veillonella*, *Streptococcus, Actinomyces*, and Lachnospiraceae *Incertae Sedis* were also associated with degradation of the same HMOs as bifidobacteria. Interestingly, high levels of degradation of 6′SL, DFL, LST a and LST c were not associated with high levels of any of the bifidobacterial OTUs, but with various OTUs belonging to *Bacteroides*, *Streptococcus*, *Varibacullum* (6′SL), *Actinomyces*, *Clostridium*, *Collinsella* and *Streptococcus* (LST c), and *Haemophilus*, *Veillonella* (DFL), and Lachnospiraceae *Incertae Sedis*, and *Halomonas* (LST a).

Negative associations were also observed for LNFP II and *Bacteroides*, and for LNFP V and *Parabacteroides* suggesting the role of these bacteria in the HMO degradation. The fact that *Bacteroides* and *Parabacteroides* (formerly also *Bacteroides*) were identified in our analysis is in line with earlier studies showing growth of *Bacteroides* spp. on selected milk glycans by activating the mucus degradation pathway^46^. Finally, LNDFH I in both milk and faeces was negatively associated with *Enterococcus* OTU 1702, but the association was stronger in faeces. Even though *in vitro* studies showed that in a monoculture *Enterococcus* was not able to grow on milk HMOs^16^, another study showed that this group was found in breastmilk^44^, that its abundance in infant faeces could be predicted from the maternal HMO profile and that it was positively correlated with the abundance of *Bifidobacterium, Streptococcus* and *Veillonella*^7^. One of the suggested explanations was that *Enterococcus* can cross feed on HMO fermentation products or HMO breakdown by-products that are released in the ecosystem by HMO degrading bifidobacteria or *Bacteroides* spp.^7^.

The correlation analysis of infant faecal HMOs and infant faecal OTUs for all time points combined also detected numerous significant positive associations between various HMOs and *Streptococcus, Staphylococcus, Escherichia-Shigella*, and *Clostridium* OTUs (Fig. 5). In both, milk and faeces LST c, 6′SL and LNnH showed significant correlation with staphylococci, while LNFP III and 3FL were positively correlated with streptococci. Earlier studies showed that neither *Streptococcus, Staphylococcus, Escherichia-Shigella*^7,16^, nor *Clostridium*^16^ could effectively utilise and grow on milk HMOs. However, all these bacterial groups are members of the microbiota of breastmilk and areolar skin^45,47,48^, and the positive link might be due to breastfeeding practises, for example with more frequent feedings resulting in higher ingested doses of the bacteria and the HMOs. If the HMOs are not well digested, the positive associations may still persist in the faeces.

Our study has few important limitations. The number of participating mother-infant pairs was relatively small and future studies should be done on larger sample sizes to verify our findings. Larger sample size would also allow for stratification of data based on maternal secretor status. Another limitation of this study is the lack of measurements of the actual volumes of the breast milk ingested and the mass of the faeces produced by each infant in a 24-hour sampling period. Therefore, our calculations had to be based on estimates from other studies that measured the daily milk intake in infants of similar ages. Actual intakes, although likely increasing over time, can vary greatly between infants, and thus, our calculated values are only an estimate and should therefore be considered as such. Measurements of volumes or weights of breastmilk samples and faecal samples should be included in future studies. Finally, with our methods we could not identify species or strains of the bacteria, nor could we quantify their actual numbers which could further support our findings.

In this study we found only weak associations between selected breastmilk HMOs and faecal microbiota community structure in breastfed infants. However, we found a strong link between changes in levels of various HMOs in infant faeces and specific microbial groups, in particular different species of *Bifidobacterium*. Earlier studies showed that different bifidobacterial species vary in their ability to break down HMOs, and some species can degrade HMOs without experiencing a detectable population growth^49,50^. Thus, including metatranscriptome or metaproteome analyses in this set would have been very helpful in understanding the community dynamics in regard of HMO metabolism in the infant GI tract. Our findings could provide the basis for assembling simple synthetic communities to study microbial interactions and community structure changes, which are centred around degradation of different HMO structures. *In vitro* fermentation studies incorporating purified compounds would also help to eliminate confounders, such as presence of milk’s own microbiota and presence of milk components, which have a regulatory effect on microbiota in both, milk and in the infant GI tract.

## Conclusion

Faecal microbiota of breastfed infants during the first 12 weeks of life is highly diverse, dynamic and influenced by age and other factors. The effect of mode of delivery disappeared after six weeks of age, whereas the effect of infant sex became detectable over time. Overall, microbiota development in this cohort followed a normal colonization pattern resulting in faecal microbial communities dominated by *Bifidobacterium*, in particular the most predominant *Bifidobacterium* OTU 1263. Breastmilk HMO analyses showed that the composition of the 18 HMOs that were measured varied between mothers and throughout the duration of lactation. In our analysis we did not observe strong and consistent positive correlations between the HMOs in maternal breastmilk and specific microbial OTUs including bifidobacteria in infants’ faeces. Thus, we believe that HMO composition is only one of many factors regulating colonization and structure of the infant GI microbial community. However, the findings of this study supported the key role of bifidobacteria in the infants’ ability to utilize most of the measured HMOs, in addition to indicating the role of other microbial taxa in the degradation or metabolism of specific HMOs.

## Methods

### Sample collection

The infants included in this study were born at term (after 37 weeks of gestation) from single pregnancies, were healthy and had no congenital abnormalities related to growth, did not receive oral or systemic antibiotic treatments, were exclusively breastfed during the study period, and had the maternal breastmilk and infant faecal samples available from each study time point. The samples originated from the BINGO (Dutch acronym for Biological Influences on Baby’s Health and Development) cohort, which is an ongoing longitudinal study investigating prenatal predictors of infant health and development. This study and all its experimental protocols were approved by and carried in accordance with the ethical committee of the Faculty of Social Sciences of the Radboud University [ECSW2014-1003-189]. Parents and/or legal guardians of all participating infants were asked to sign an informed consent form and were free to withdraw from the study at any point. The study design and infant recruitment criteria can be found at http://www.bingo-onderzoek.nl/deelname/. Both, the infant faecal samples and the maternal breastmilk samples were collected between years 2015–2016, within a 48-hour period, by the mothers at home, at two, six, and 12 weeks post-partum. Breastmilk samples (approximately 20 ml) were collected in the morning before feeding the infant, into clean, sterile collection cups as previously described^51^. Mothers were asked to wash hands, breasts and nipples before collecting the sample by hand, or in case of mechanical collection, to first sterilize the breast pump compartments by boiling. Stool samples were collected from infants’ diapers using sterile stool collection vials (80 × 16.5 mm; cat#:80.623.022, Sarstedt; Nümbrecht, Germany). Mothers were asked to save all faecal sample up to one-third of the vial. Milk and faecal samples were stored by the participants in their home freezers until collected by the experimenter within a week after the last collection time point. Subsequently all milk and faecal samples were stored at −80 °C until further processing and analysis. Samples were analysed for breastmilk HMOs, corresponding faecal HMOs, and microbiota composition.

### HMO analysis in breastmilk and faeces

Eighteen different HMO structures were analysed in milk and infant faeces, including 13 neutral HMOs (2′FL, 3FL, DFL, LNDFH I, LNDFH II, LNFP I, LNFP II, LNFP III, LNFP V, LNH, LNnH, pLNH, LNTandLNnT) and five acidic HMOs (3′SL, 6′SL, LST a, LST b, LST c) (Table S1). The HMOs were extracted, purified by solid phase extraction (SPE), and quantified by using porous graphitized carbon-ultra high-performance liquid chromatography - mass spectrometry (PGC-UPLC-MS)^52–54^, or by high performance anion exchange chromatography with pulsed amperometric detection (HPAEC-PAD)^55^.

### DNA extraction, amplification of 16S rRNA genes and sequencing

Total bacterial DNA was extracted from 0.1–0.15 g of faeces using the double bead-beating procedure and the Maxwell® 16 Total RNA system (Promega; Wisconsin, USA) customized with Stool Transport and Recovery Buffer (STAR; Roche Diagnostics Corporation, Indianapolis, IN) for the use with the DNA samples, as previously described^56^. The resulting DNA templates (20 ng) were used for subsequent PCR amplification of the V4 region of 16S ribosomal RNA (rRNA) genes using barcoded primers 515F-n (5′-GTGCCAGCMGCCGCGGTAA-) and 806R-n (5′-GGACTACHVGGGTWTCTAAT) following conditions described previously^56^. Seventy unique barcode tags were used in each library^57^. Negative control blanks were included during DNA extraction and PCR to check for any possible contamination. The negative controls samples gave no PCR products when visualised on agarose gels, and subsequently were not sequenced. All remaining PCR products were purified, 100 ng of each barcoded sample was added to an amplicon pool and the pools were adjusted to 100 ng/µL final concentration. The libraries were sent for adapter ligation and Illumina HiSeq 2000 sequencing at GATC-Biotech, Konstanz, Germany^56^.

### Data analysis

The 16S rRNA sequencing data analysis was carried out using the NG-Tax analysis pipeline with standard parameters^57^. Filtered libraries contained only read pairs with perfectly matching barcodes that were subsequently used to separate reads by sample. OTUs were assigned using an open reference approach and the SILVA_111_SSU 16S rRNA gene reference database (https://www.arb-silva.de/)^58^. Microbial composition data was expressed as a relative abundance of each OTU obtained with NG-Tax.

### Statistical analyses

Milk and faecal HMOs concentrations were measured in µg per mL of milk or µg per gram of faeces. Readout values were normalised for each time point separately around mean using the Probabilistic Quotient Normalization (PQN) method in R (version 3.3.2) to correct for sample to sample variability due to naturally occurring differences in milk dilutions. Changes in HMO concentrations between study time points were assessed using one-way repeated measures ANOVA for HMOs for normally distributed data sets as assessed with Shapiro-Wilk’s normality test. Differences between not-normally distributed HMO sets were tested using nonparametric Kruskal-Wallis test. We estimated average daily volumes of ingested breastmilk based on literature data^19^ to be 480 g at week two, 580 g at week six, and 630 g at week 12 of life. These values were then used to calculate the daily amount of each HMO consumed by infants in our study at each time point.

Microbial composition data was expressed as relative abundance (RA) of each OTU obtained in the NG-Tax pipeline. OTUs which had a prevalence of less than 5% across all samples were removed and their values were summarized as “Other OTUs”. Alpha diversity index determinations (Shannon, Chao1, and PD Whole Tree) were carried out in QIIME on rarefied data with OTU cut-off of 3380 reads per sample^59^. Spearman correlations were calculated using R to evaluate associations between OTU members of the faecal microbial community, and between the faecal OTUs and milk or faecal HMO concentrations. We only reported associations which passed the correlation threshold of ±0.3 and significance of p < 0.05. Unconstrained (PCA) and constrained (RDA) multivariate analyses were carried out in Canoco5 on log transformed HMO concentrations data and on microbial OTU level relative abundance data with the significance assessed using the Monte Carlo permutation test at 499 random permutations^60^. For better image clarity, only the top twenty best fitting taxa were displayed on the PCA and RDA plots. These taxa were those for which the highest percentage of variation in the relative abundance data was explained by the ordination axes. The vectors corresponding to these taxa point towards the ordination plane where samples containing higher relative abundance of these taxa are located and the lengths of these vectors equal to R- squared calculated by dividing the taxa scores by their SD^60^. The explanatory variables used in the RDA included infant age at the time of collection, estimated amounts of the 18 milk HMO ingested during a 24 h period (mg/24 h) or faecal HMOs (µg/g of faeces), infant sex, place and mode of delivery, maternal secretor status, and if an infant was sick at the time of sample collection, as recalled by the mother. Degradation of each breastmilk HMO was calculated as a ratio of HMO concentration in infants’ faeces and the concentration of the same HMO measured in mothers’ milk. If a given HMO was not detected in milk, the consumption score was not included in the analysis, and if the concentration in faeces exceeded the amount detected in milk, the infant was assigned to the “low” category for that HMO. Resulting values (ratios) and the natural breaks in the data were then used for separating and assign infants to either a “low”, “medium”, or a “high” consumption category for each HMO at each time point. The association between faecal microbiota composition and the assignment of each infant to a “low”, “medium”, or “high” consumption category for each HMO were investigated with RDA in Canoco5, with significance assessed using a permutation test^60^. Differentially abundant OTUs were then identified by comparing the microbial abundance data of infants assigned into “high” and “low” consumption groups and for each individual HMO using Kruskal-Wallis analysis in QIIME with a significance cut-off set at FDR < 0.05^59^.

### Nucleotide sequences

BINGO data sets cannot be made publicly available due to the data being part of an ongoing longitudinal study. Parts of the data are available to the research community for scientific collaborations upon request to Prof. Dr. C. de Weerth at: Department of Cognitive Neuroscience, Donders Institute for Brain, Cognition and Behaviour, Radboud University Medical Center, 6525 HR Nijmegen, the Netherlands, e-mail: Carolina.deWeerth@radboudumc.nl.

## Supplementary information

Supplementary Figures and Tables.

## References

[1] Koenig JE (2011). Succession of microbial consortia in the developing infant gut microbiome. Proc. Natl. Acad. Sci. USA.

[2] Zivkovic AM, Lewis ZT, German B, Mills DA (2013). Establishment of a milk-oriented microbiota (MOM) in early life: how babies meet their MOMs. Funct. Food. Rev..

[3] Chu DM (2017). Maturation of the infant microbiome community structure and function across multiple body sites and in relation to mode of delivery. Nat. Med..

[4] Kummeling I (2005). Etiology of atopy in infancy: the KOALA Birth Cohort Study. Pediatr. Allergy. Immunol..

[5] Scheepers LE (2015). The intestinal microbiota composition and weight development in children: the KOALA Birth Cohort Study. Int. J. Obes..

[6] Mueller NT, Bakacs E, Combellick J, Grigoryan Z, Dominguez-Bello MG (2015). The infant microbiome development: mom matters. Trends Mol. Med..

[7] Wang M (2015). Fecal microbiota composition of breast-fed infants is correlated with human milk oligosaccharides consumed. J. Pediatr. Gastroenterol. Nutr..

[8] Coppa GV (1993). Changes in carbohydrate composition in human milk over 4 months of lactation. Pediatrics.

[9] Austin S (2016). Temporal change of the content of 10 oligosaccharides in the milk of Chinese urban mothers. Nutrients.

[10] Sprenger N, Lee LY, De Castro CA, Steenhout P, Thakkar SK (2017). Longitudinal change of selected human milk oligosaccharides and association to infants’ growth, an observatory, single center, longitudinal cohort study. PLOS One.

[11] Bode L (2012). Human milk oligosaccharides: every baby needs a sugar mama. Glycobiology.

[12] Azad MB (2018). Human milk oligosaccharide concentrations are associated with multiple fixed and modifiable maternal characteristics, environmental factors, and feeding practices. J. Nutr..

[13] Smilowitz JT, Lebrilla CB, Mills DA, German JB, Freeman SL (2014). Breast milk oligosaccharides: structure-function relationships in the neonate. Annu. Rev. Nutr..

[14] Albrecht S, Schols HA, van den Heuvel EGHM, Voragen AGJ, Gruppen H (2011). Occurrence of oligosaccharides in feces of breast-fed babies in their first six months of life and the corresponding breast milk. Carbohyd. Res..

[15] Albrecht S (2011). Oligosaccharides in feces of breast- and formula-fed babies. Carbohyd. Res..

[16] Marcobal A (2010). Consumption of human milk oligosaccharides by gut-related microbes. J. Agric. Food. Chem..

[17] Hong Q (2014). Label-free absolute quantitation of oligosaccharides using multiple reaction monitoring. Anal. Chem..

[18] Ceroni A (2007). GlycoWorkbench: A Tool for the Computer-Assisted Annotation of Mass Spectra of Glycans. J. Proteome Res..

[19] Institute of Medicine. Nutrition during lactation. 5, Milk volume. Nat. Acad. Press, 1–326, 10.17226/1577 (1991).

[20] Yatsunenko T (2012). Human gut microbiome viewed across age and geography. Nature.

[21] Scholtens PA, Oozeer R, Martin R, Amor KB, Knol J (2012). The early settlers: intestinal microbiology in early life. Ann. Rev. Food Sci. Technol..

[22] Timmerman HM (2017). Intestinal colonisation patterns in breastfed and formula-fed infants during the first 12 weeks of life reveal sequential microbiota signatures. Sci. Rep..

[23] Bäckhed F (2015). Dynamics and stabilization of the human gut microbiome during the first year of life. Cell Host Microbe.

[24] Biasucci G (2010). Mode of delivery affects the bacterial community in the newborn gut. Early. Hum. Dev..

[25] Rutayisire E, Huang K, Liu Y, Tao F (2016). The mode of delivery affects the diversity and colonization pattern of the gut microbiota during the first year of infants’ life: a systematic review. BMC Gastroenterol..

[26] Black M, Bhattacharya S, Philip S, Norman JE, McLernon DJ (2015). Planned cesarean delivery at term and adverse outcomes in childhood health. JAMA.

[27] Kuhle S, Tong OS, Woolcott CG (2015). Association between caesarean section and childhood obesity: a systematic review and meta-analysis. Obes. Rev..

[28] Fransen, F. *et al*. The impact of gut microbiota on gender-specific differences in immunity. *Front. Immunol*. **8**, 10.3389/fimmu.2017.00754 (2017). 10.3389/fimmu.2017.00754 PMC5491612 28713378

[29] Mueller S (2006). Differences in fecal microbiota in different European study populations in relation to age, gender, and country: a cross-sectional study. Appl. Environ. Microbiol..

[30] Cong X (2016). Gut microbiome developmental patterns in early life of preterm infants: impacts of feeding and gender. PLOS One.

[31] Martin R (2016). Early-life events, including mode of delivery and type of feeding, siblings and gender, shape the developing gut microbiota. PLOS ONE.

[32] Butte NF, Wong WW, Hopkinson JM, O’Brian Smith E, Ellis KJ (2000). Infant feeding mode affects early growth and body composition. Pediatrics.

[33] Abrahamsson TR (2014). Low gut microbiota diversity in early infancy precedes asthma at school age. Clin. Exp. Allergy..

[34] Harmsen HJM (2000). Analysis of intestinal flora development in breast-fed and formula-fed infants by using molecular identification and detection methods. J. Ped. Gastroenterol. Nutr..

[35] Kleessen, B., Bunke, H., Tovar, K., Noack, J. & Sawatzki, G. Influence of two infant formulas and human milk on the development of the faecal flora in newborn infants. Acta Pediatr., 1347–1356 (1995). 10.1111/j.1651-2227.1995.tb13567.x 8645949

[36] Kunz C, Rudloff S (1993). Biological functions of oligosaccharides in human milk. Acta Pediatr..

[37] Stark P, Lee A (1982). The microbial ecology of the large bowel of breast-fed and formula-fed infants during the first year of life. J. Med. Microbiol..

[38] Balmer S, Wharton B (1989). Diet and faecal flora in the newborn: breast milk and infant formula. Arch. Dis. Child..

[39] Asakuma S (2011). Physiology of consumption of human milk oligosaccharides by infant gut-associated bifidobacteria. J. Biol. Chem..

[40] Coppa GV (2006). Human milk oligosaccharides inhibit the adhesion to Caco-2 cells of diarrheal pathogens: Escherichia coli, Vibrio cholerae, and Salmonella fyris. Pediatr. Res..

[41] Jantscher-Krenn E (2012). Human milk oligosaccharides reduce Entamoeba histolytica attachment and cytotoxicity *in vitro*. Br. J. Nutr..

[42] Guo Y (2004). Structural basis for distinct ligand-binding and targeting properties of the receptors DC-SIGN and DC-SIGNR. Nat. Struct. Mol. Biol..

[43] Moossavi S (2019). Composition and variation of the human milk microbiota are influenced by maternal and early-life factors. Cell Host Microbe.

[44] Aakko J (2017). Human milk oligosaccharide categories define the microbiota composition in human colostrum. Benef. Microbes.

[45] Pannaraj PS (2017). Association between breast milk bacterial communities and establishment and development of the infant gut microbiome. JAMA Pediatr..

[46] Marcobal A (2011). Bacteroides in the infant gut consume milk oligosaccharides via mucus-utilization pathways. Cell Host Microbe.

[47] Hunt KM (2011). Characterization of the diversity and temporal stability of bacterial communities in human milk. PLOS One.

[48] Jost T, Lacroix C, Braegger CP, Rochat F, Chassard C (2014). Vertical mother-neonate transfer of maternal gut bacteria via breastfeeding. Environ. Microbiol..

[49] Kiyohara M (2011). An exo-alpha-sialidase from bifidobacteria involved in the degradation of sialyloligosaccharides in human milk and intestinal glycoconjugates. Glycobiology.

[50] Ward RE, Ninonuevo M, Mills DA, Lebrilla CB, German JB (2007). *In vitro* fermentability of human milk oligosaccharides by several strains of bifidobacteria. Mol. Nutr. Food. Res..

[51] Hechler C, Beijers R, Riksen-Walraven JM, de Weerth C (2018). Are cortisol concentrations in human breast milk associated with infant crying?. Dev. Psychobiol..

[52] Albrecht S, Schols HA, van den Heuvel EG, Voragen AG, Gruppen H (2010). CE-LIF-MS n profiling of oligosaccharides in human milk and feces of breast-fed babies. Electrophoresis.

[53] Wu S, Tao N, German JB, Grimm R, Lebrilla CB (2010). Development of an annotated library of neutral human milk oligosaccharides. J. Proteome Res..

[54] Wu S, Grimm R, German JB, Lebrilla CB (2011). Annotation and structural analysis of sialylated human milk oligosaccharides. J. Proteome Res..

[55] Thurl S, Muller-Werner B, Sawatzki G (1996). Quantification of individual oligosaccharide compounds from human milk using high-pH anion-exchange chromatography. Anal. Biochem..

[56] Gu, F. *et al*. *In vitro* fermentation behavior of isomalto/malto-polysaccharides using human fecal inoculum indicates prebiotic potential. *Mol. Nutr. Food Res*., 10.1002/mnfr.201800232 (2018). 10.1002/mnfr.201800232 PMC6033187 29710405

[57] Ramiro-Garcia J (2016). NG-Tax, a highly accurate and validated pipeline for analysis of 16S rRNA amplicons from complex biomes. F1000Research.

[58] Quast C (2013). The SILVA ribosomal RNA gene database project: improved data processing and web-based tools. Nucleic Acids Res..

[59] Caporaso, J. G. *et al*. QIIME allows analysis of high-throughput community sequencing data. *Nat. Methods*, 10.1038/nmeth0510-335 (2010). 10.1038/nmeth.f.303 PMC3156573 20383131

[60] Šmilauer, P. & Lepš, J. Multivariate analysis of ecological data using CANOCO 5. Cambridge University Press, 10.1017/CBO9781139627061 (2014).

