# Supplementary material for: The association between breastmilk oligosaccharides and faecal microbiota in healthy breastfed infants at two, six, and twelve weeks of age
Source: Sci Rep. 2020 Mar 6;10:4270. doi: 10.1038/s41598-020-61024-z (PMC7060319; doi:10.1038/s41598-020-61024-z)
Supplement: Supplementary file 1 — Supplementary Figures and Tables. [file 41598_2020_61024_MOESM1_ESM.docx]

**Supplementary information for the manuscript entitled:**

***The association between breastmilk oligosaccharides and faecal microbiota in healthy breastfed infants at two, six, and twelve weeks of age***

Klaudyna Borewicz, Fangjie Gu, Edoardo Saccenti, Christine Hechler, Roseriet Beijers, Carolina de Weerth, Sander S. van Leeuwen, Henk A. Schols, Hauke Smidt

**
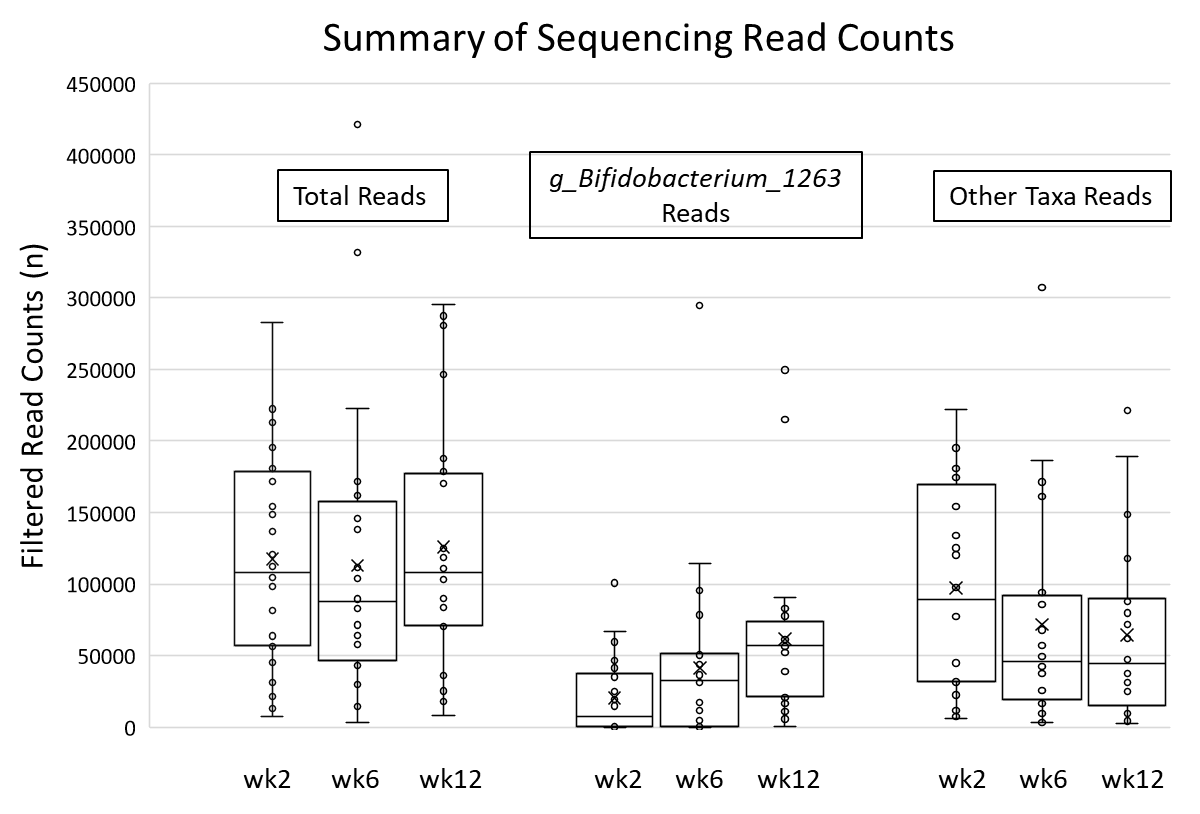
**

**Figure S1.** Box and whisker plots showing the minimum, first quartile, median, third quartile, and maximum number of the filtered sequencing reads from infants’ faecal samples at two, six, and 12 week collection timepoints. Total number of filtered reads, a subset of reads that were assigned to *g_Bifidobacterium_1263*, and a sum of reads from all other taxa are displayed.


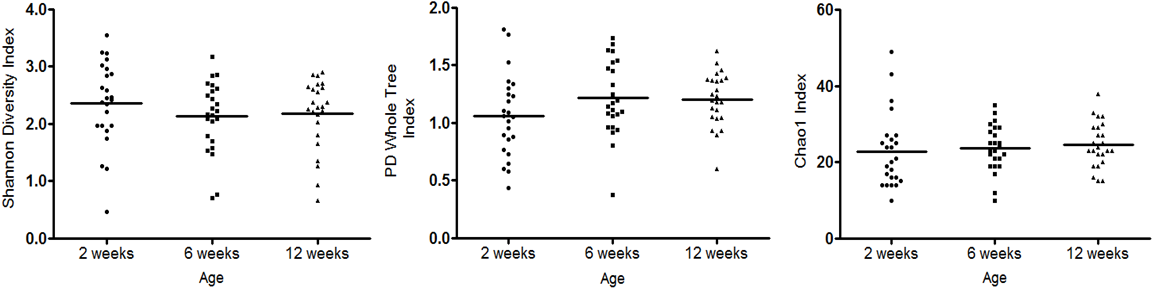


**Figure S2.** Alpha diversity indices at different time points. Diversity was estimated using Shannon diversity index, which evaluates both the number of species and the evenness of their distribution. Richness, which estimates the number of different species present in each sample, was measured with the Chao1 Index and with PD Whole Tree, the latter of which takes into account phylogenetic differences between species (OTUs). No significant differences were found between any of the age groups.

a.


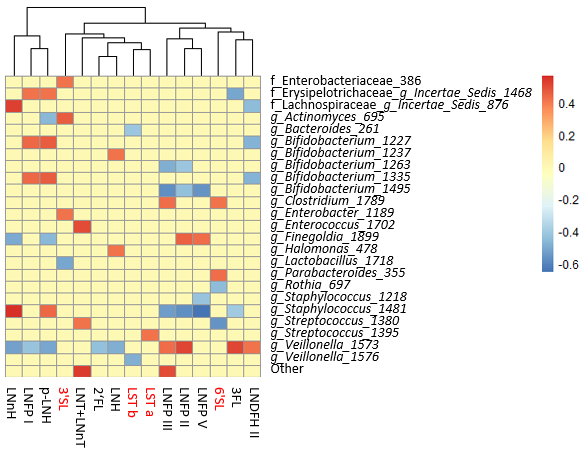


b.


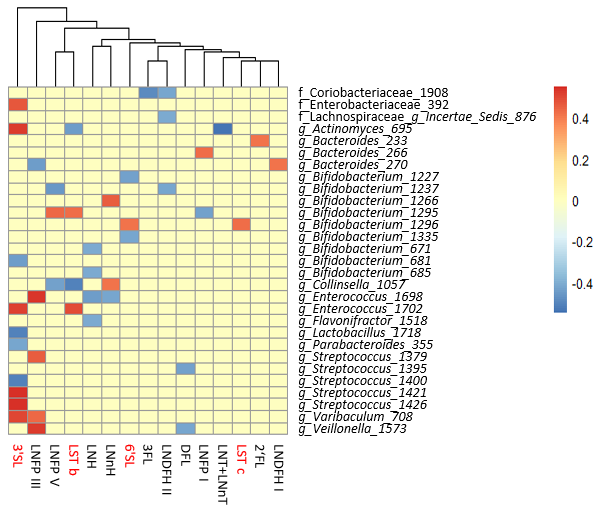


c.


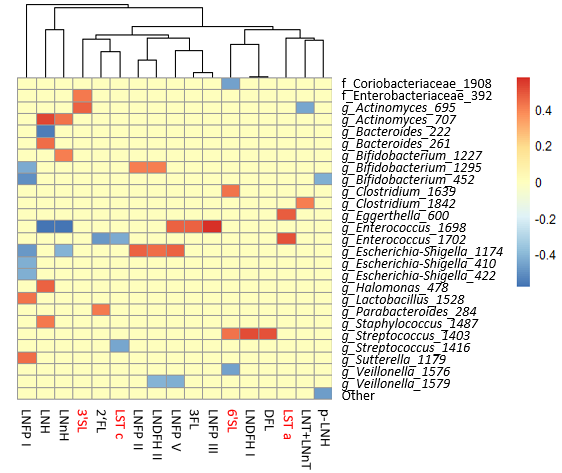


**Figure S3.** Statistically significant (p<0.05) Spearman correlations (correlation threshold ±0.3) between an estimated daily intake of different breastmilk HMOs and faecal microbiota composition at OTU level of 24 infants at two (a), six (b) and 12 (c) weeks after birth. Positive associations are indicated in red, negative in blue, yellow denotes correlations that did not pass the significance or the correlation thresholds. The names of acidic HMOs are highlighted in red.

a.


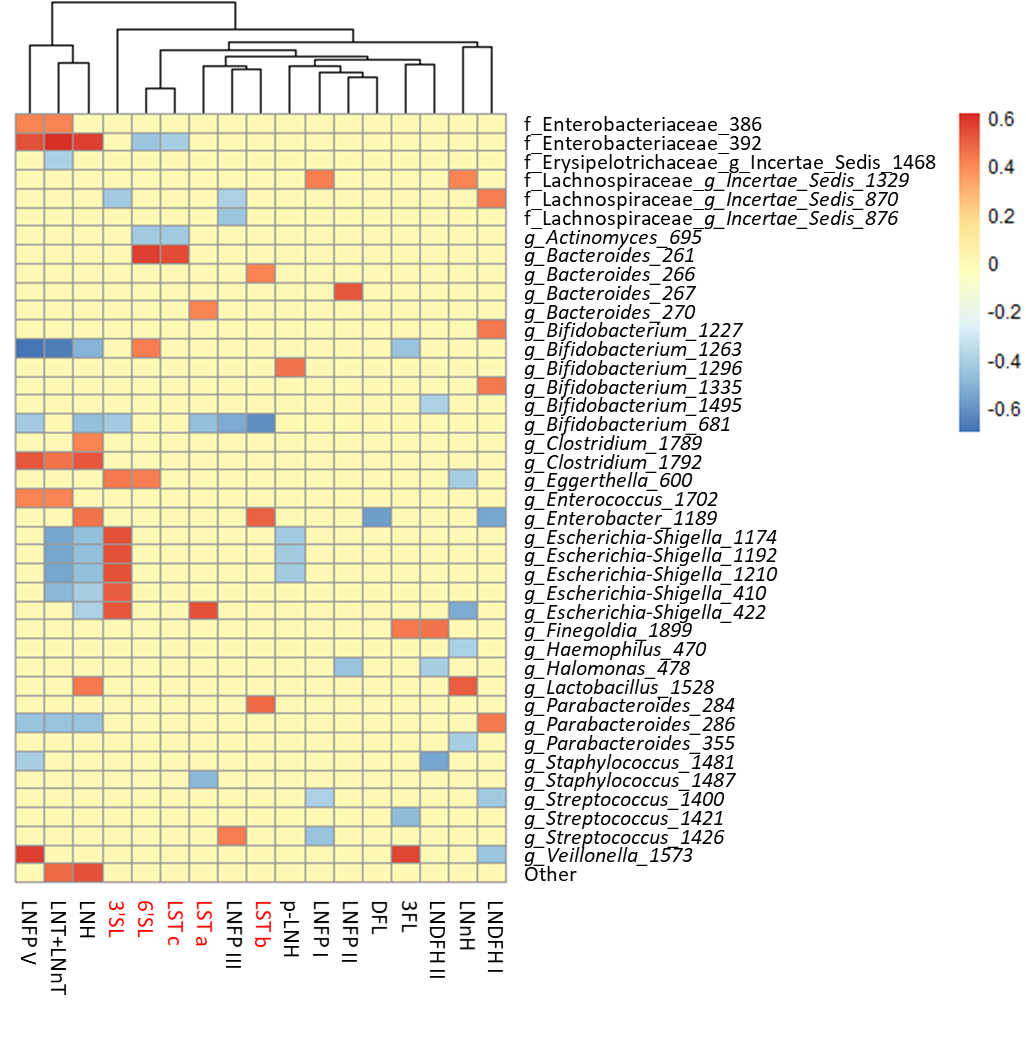


b.


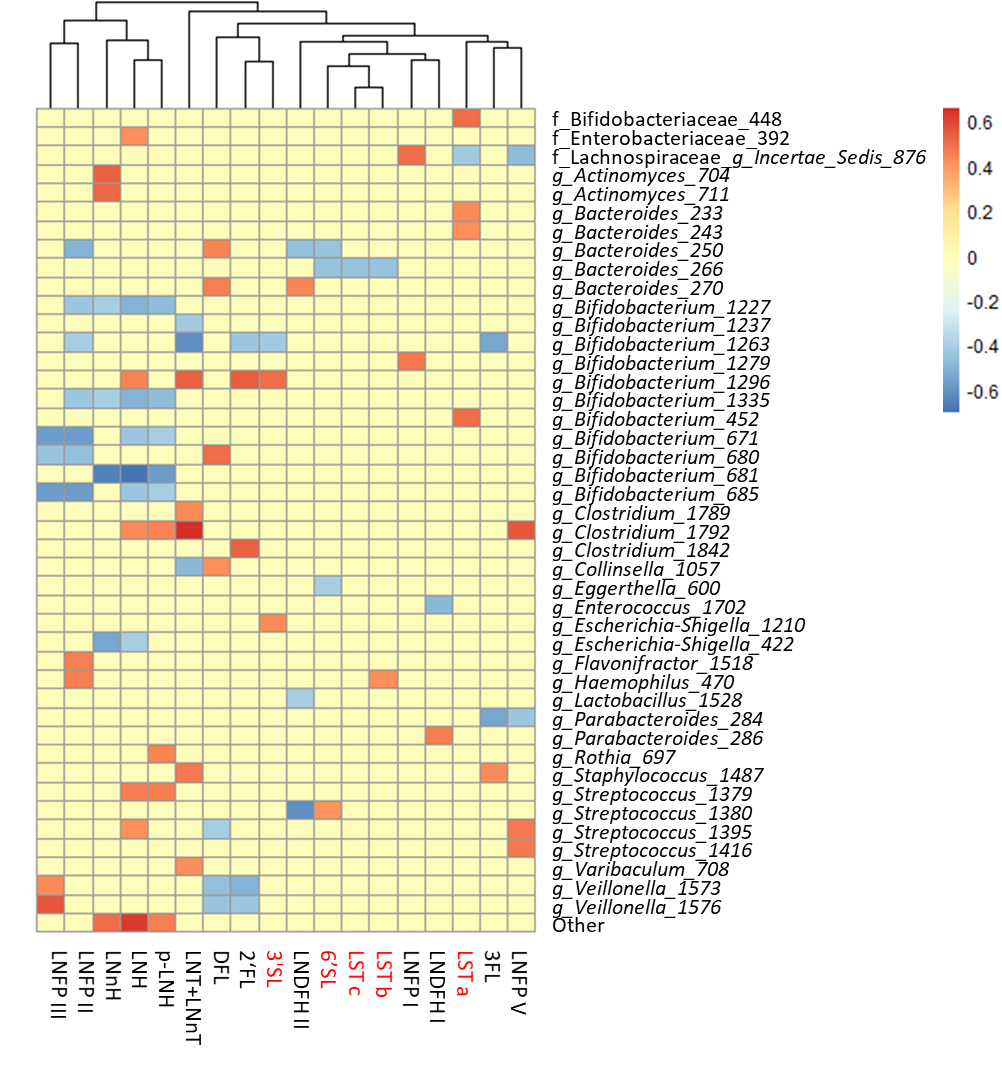


c.


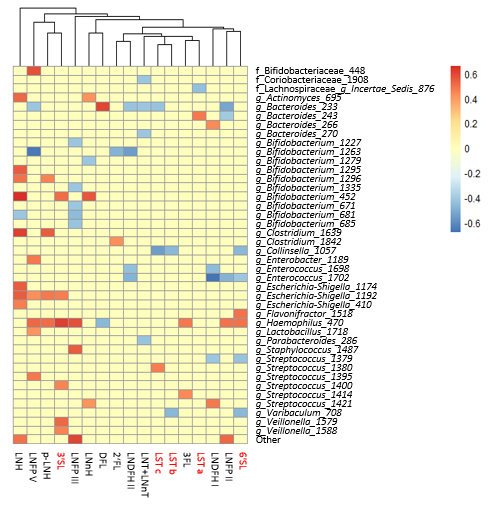


Figure S4. Statistically significant (p<0.05) Spearman correlations (correlation threshold ±0.3) between different HMOs detected in infant faeces and faecal microbiota composition at OTU level of 24 infants at two (a), six (b) and 12 (c) weeks after birth. Positive associations are indicated in red, negative in blue, and correlations that did not pass the significance or the correlation thresholds are marked in yellow. The names of acidic HMOs are highlighted in red.

Table S1. HMO categories, names and structures (51) included in this study. Blue circle – glucose; yellow circle – galactose; blue square N – acetylglucosamine; red triangle – fucose; purple diamond – sialic acid

| Category | Name | Abbreviation | Structure |
| --- | --- | --- | --- |
| Neutral | 3-fucosyllactose | 3FL | 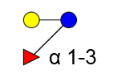 |
|  | 2'-fucosyllactose | 2'FL | 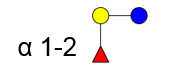 |
|  | Lacto-*N*-tetraose | LNT | 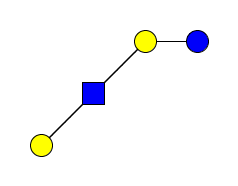 |
|  | Lacto-*N*-neotetraose | LNnT | 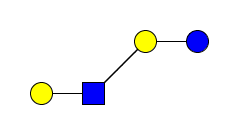 |
|  | Lacto-*N*-fucopentaose I | LNFP I | 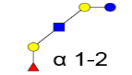 |
|  | Lacto-*N*-fucopentaose II | LNFP II | 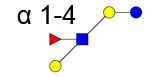 |
|  | Lacto-*N*-fucopentaose III | LNFP III | 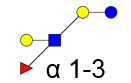 |
|  | Lacto-*N*-fucopentaose V | LNFP V | 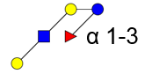 |
|  | Difucosyllactose | DFL | 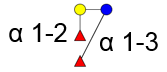 |
|  | Lacto-*N*-difucohexaose I | LNDFH I | 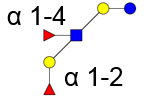 |
|  | Lacto-*N*-difucohexaose II | LNDFH II | 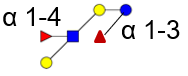 |
|  | Para-lacto-*N*- hexaose | pLNH | 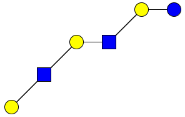 |
|  | Lacto-*N*-hexaose | LNH | 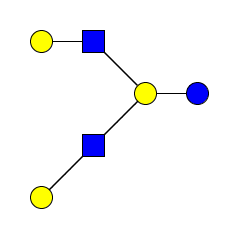 |
|  | Lacto-*N*-neohexaose | LNnH | 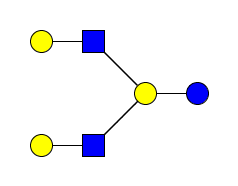 |
| Acidic | 6'-sialyllactose | 6'SL | 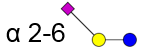 |
|  | 3'-sialyllactose | 3'SL | 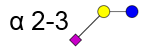 |
|  | Sialyl-lacto-*N*-tetraose a | LST a | 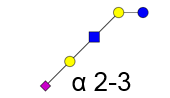 |
|  | Sialyl-lacto-*N*-tetraose b | LST b | 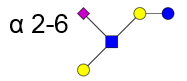 |
|  | Sialyl-lacto-*N*-tetraose c | LST c | 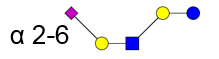 |

Table S2. Average relative abundance (Avg RA), standard error of abundance means (SEM), and OTU prevalence of OTUs (n=411) at two, six and 12 weeks of age. Only OTUs which were present in at least 5% of all samples are shown

| **Genus** | **OTU#** | **Week two (n=24)** | | | **Week six (n=24)** | | | **Week 12 (n=24)** | | | **Total (n=72)** | | |
| --- | --- | --- | --- | --- | --- | --- | --- | --- | --- | --- | --- | --- | --- |
|  |  | **Avg RA (%)** | **± SEM** | **Prevelance (%)** | **Avg RA (%)** | **± SEM** | **Prevelance (%)** | **Avg RA (%)** | **± SEM** | **Prevelance (%)** | **Avg RA (%)** | **± SEM** | **Prevelance (%)** |
| *Actinomyces* | 695 | 0.89 | 0.88 | 8.33 | 2.17 | 2.16 | 12.5 | 0.17 | 0.16 | 8.33 | 1.08 | 0.77 | 9.72 |
|  | 704 | 0.3 | 0.28 | 12.5 | 0.06 | 0.04 | 12.5 | 0.01 | 0.01 | 4.17 | 0.12 | 0.09 | 9.72 |
|  | 707 | 0 | 0 | 0 | 0.01 | 0.01 | 4.17 | 0.07 | 0.03 | 20.83 | 0.03 | 0.01 | 8.33 |
|  | 711 | 0 | 0 | 0 | 0.07 | 0.05 | 8.33 | 0.12 | 0.08 | 16.67 | 0.06 | 0.03 | 8.33 |
| *Aeribacillus* | 1603 | 0.01 | 0.01 | 4.17 | 0.02 | 0.01 | 12.5 | 0.01 | 0.01 | 4.17 | 0.01 | 0 | 6.94 |
| *Bacteroides* | 222 | 2.67 | 1.17 | 33.33 | 7.97 | 3.24 | 50 | 5.48 | 2.16 | 50 | 5.37 | 1.36 | 44.44 |
|  | 233 | 0.09 | 0.08 | 8.33 | 0.04 | 0.02 | 16.67 | 0.1 | 0.04 | 25 | 0.07 | 0.03 | 16.67 |
|  | 243 | 0.03 | 0.03 | 8.33 | 0.07 | 0.05 | 8.33 | 0.39 | 0.2 | 20.83 | 0.16 | 0.07 | 12.5 |
|  | 250 | 0.05 | 0.04 | 8.33 | 0.07 | 0.05 | 12.5 | 0.23 | 0.12 | 16.67 | 0.12 | 0.04 | 12.5 |
|  | 261 | 1.31 | 1.29 | 12.5 | 0.07 | 0.07 | 4.17 | 0.04 | 0.02 | 12.5 | 0.47 | 0.43 | 9.72 |
|  | 266 | 0.36 | 0.28 | 8.33 | 0.08 | 0.05 | 12.5 | 0.02 | 0.01 | 8.33 | 0.15 | 0.1 | 9.72 |
|  | 267 | 0.5 | 0.4 | 12.5 | 0.27 | 0.19 | 16.67 | 0.1 | 0.08 | 12.5 | 0.29 | 0.15 | 13.89 |
|  | 270 | 0.32 | 0.29 | 12.5 | 0.08 | 0.04 | 16.67 | 0.07 | 0.04 | 12.5 | 0.16 | 0.1 | 13.89 |
| *Bifidobacterium* | 1227 | 0.11 | 0.08 | 8.33 | 0.14 | 0.07 | 20.83 | 0.28 | 0.1 | 37.5 | 0.17 | 0.05 | 22.22 |
|  | 1237 | 0.13 | 0.09 | 8.33 | 0.17 | 0.1 | 16.67 | 0.21 | 0.14 | 25 | 0.17 | 0.06 | 16.67 |
|  | 1263 | 22.23 | 5.39 | 83.33 | 35.1 | 6.23 | 91.67 | 51.26 | 4.96 | 100 | 36.2 | 3.46 | 91.67 |
|  | 1265 | 0.04 | 0.02 | 12.5 | 0.01 | 0.01 | 4.17 | 0.01 | 0.01 | 4.17 | 0.02 | 0.01 | 6.94 |
|  | 1266 | 0.01 | 0.01 | 4.17 | 0.02 | 0.01 | 8.33 | 0.03 | 0.02 | 8.33 | 0.02 | 0.01 | 6.94 |
|  | 1279 | 11.92 | 3.18 | 58.33 | 11.93 | 3.82 | 79.17 | 6.01 | 2.86 | 79.17 | 9.95 | 1.91 | 72.22 |
|  | 1295 | 0.05 | 0.03 | 16.67 | 0.06 | 0.03 | 20.83 | 0.07 | 0.03 | 20.83 | 0.06 | 0.02 | 19.44 |
|  | 1296 | 0.07 | 0.04 | 16.67 | 0.05 | 0.03 | 12.5 | 0.02 | 0.02 | 4.17 | 0.05 | 0.02 | 11.11 |
|  | 1335 | 0.09 | 0.06 | 8.33 | 0.12 | 0.06 | 20.83 | 0.24 | 0.08 | 37.5 | 0.15 | 0.04 | 22.22 |
|  | 1495 | 0.06 | 0.03 | 16.67 | 0 | 0 | 0 | 0 | 0 | 0 | 0.02 | 0.01 | 5.56 |
|  | 452 | 0.02 | 0.02 | 4.17 | 0.02 | 0.01 | 8.33 | 0.05 | 0.02 | 20.83 | 0.03 | 0.01 | 11.11 |
|  | 671 | 0.03 | 0.03 | 4.17 | 0.08 | 0.04 | 16.67 | 0.24 | 0.08 | 37.5 | 0.12 | 0.03 | 19.44 |
|  | 680 | 0.01 | 0.01 | 4.17 | 0.02 | 0.01 | 12.5 | 0.09 | 0.04 | 25 | 0.04 | 0.01 | 13.89 |
|  | 681 | 5.92 | 3.51 | 16.67 | 9.46 | 4.41 | 41.67 | 6.48 | 2.38 | 58.33 | 7.29 | 2.02 | 38.89 |
|  | 685 | 0.03 | 0.03 | 4.17 | 0.08 | 0.04 | 16.67 | 0.28 | 0.1 | 37.5 | 0.13 | 0.04 | 19.44 |
| *Clostridium* | 1629 | 0.02 | 0.02 | 4.17 | 0.52 | 0.52 | 4.17 | 0.18 | 0.18 | 4.17 | 0.24 | 0.18 | 4.17 |
|  | 1639 | 0.03 | 0.03 | 4.17 | 0.04 | 0.04 | 4.17 | 0.28 | 0.2 | 8.33 | 0.12 | 0.07 | 5.56 |
|  | 1789 | 0.06 | 0.03 | 12.5 | 0.02 | 0.01 | 8.33 | 0 | 0 | 0 | 0.03 | 0.01 | 6.94 |
|  | 1792 | 1.87 | 0.84 | 20.83 | 0.79 | 0.37 | 45.83 | 0.32 | 0.15 | 25 | 0.99 | 0.31 | 30.56 |
|  | 1842 | 0 | 0 | 0 | 0.08 | 0.07 | 12.5 | 0.08 | 0.07 | 8.33 | 0.06 | 0.03 | 6.94 |
| *Collinsella* | 1057 | 0.05 | 0.05 | 4.17 | 0.09 | 0.06 | 12.5 | 0.12 | 0.07 | 16.67 | 0.09 | 0.03 | 11.11 |
| *Eggerthella* | 600 | 0.09 | 0.05 | 12.5 | 0.14 | 0.08 | 20.83 | 0.09 | 0.06 | 12.5 | 0.1 | 0.04 | 15.28 |
| *Enterobacter* | 1189 | 0.02 | 0.02 | 8.33 | 0.01 | 0.01 | 4.17 | 0.02 | 0.01 | 8.33 | 0.02 | 0.01 | 6.94 |
| *Enterococcus* | 1698 | 0.01 | 0.01 | 4.17 | 0.15 | 0.1 | 12.5 | 0.18 | 0.1 | 20.83 | 0.11 | 0.05 | 12.5 |
|  | 1702 | 0.48 | 0.25 | 25 | 0.77 | 0.35 | 37.5 | 0.84 | 0.3 | 54.17 | 0.7 | 0.17 | 38.89 |
| *Escherichia-Shigella* | 1174 | 0.04 | 0.02 | 16.67 | 0.02 | 0.01 | 12.5 | 0.06 | 0.03 | 20.83 | 0.04 | 0.01 | 16.67 |
|  | 1192 | 0.04 | 0.02 | 16.67 | 0.01 | 0.01 | 4.17 | 0.02 | 0.02 | 8.33 | 0.02 | 0.01 | 9.72 |
|  | 1210 | 0.04 | 0.02 | 16.67 | 0.03 | 0.02 | 16.67 | 0.05 | 0.02 | 16.67 | 0.04 | 0.01 | 16.67 |
|  | 410 | 0.12 | 0.05 | 29.17 | 0.08 | 0.03 | 29.17 | 0.17 | 0.05 | 50 | 0.12 | 0.02 | 36.11 |
|  | 422 | 8.45 | 3.68 | 66.67 | 4.42 | 1.58 | 58.33 | 5.09 | 1.46 | 75 | 5.99 | 1.42 | 66.67 |
| *Finegoldia* | 1899 | 0.06 | 0.05 | 12.5 | 0.06 | 0.06 | 4.17 | 0.01 | 0.01 | 4.17 | 0.04 | 0.03 | 6.94 |
| *Flavonifractor* | 1518 | 0 | 0 | 0 | 0.02 | 0.01 | 8.33 | 0.04 | 0.03 | 8.33 | 0.02 | 0.01 | 5.56 |
| *Gemella* | 1606 | 0.01 | 0.01 | 4.17 | 0.01 | 0.01 | 4.17 | 0.02 | 0.01 | 8.33 | 0.01 | 0.01 | 5.56 |
| *Haemophilus* | 470 | 0.18 | 0.08 | 25 | 0.32 | 0.15 | 37.5 | 0.7 | 0.41 | 41.67 | 0.4 | 0.15 | 34.72 |
| *Halomonas* | 478 | 0.01 | 0.01 | 8.33 | 0.03 | 0.02 | 12.5 | 0.01 | 0.01 | 8.33 | 0.02 | 0.01 | 9.72 |
| *Lactobacillus* | 1205 | 0 | 0 | 0 | 0.01 | 0.01 | 4.17 | 0.04 | 0.02 | 16.67 | 0.01 | 0.01 | 6.94 |
|  | 1334 | 0.01 | 0.01 | 4.17 | 0.01 | 0.01 | 4.17 | 0.03 | 0.02 | 12.5 | 0.02 | 0.01 | 6.94 |
|  | 1528 | 0.55 | 0.31 | 20.83 | 1.28 | 0.41 | 41.67 | 1.7 | 0.51 | 50 | 1.18 | 0.25 | 37.5 |
|  | 1718 | 0.46 | 0.32 | 12.5 | 0.71 | 0.34 | 25 | 0.04 | 0.03 | 8.33 | 0.4 | 0.16 | 15.28 |
| *Parabacteroides* | 284 | 0.19 | 0.14 | 12.5 | 0.12 | 0.09 | 16.67 | 0.04 | 0.03 | 8.33 | 0.12 | 0.06 | 12.5 |
|  | 286 | 0.34 | 0.17 | 29.17 | 0.72 | 0.31 | 29.17 | 0.47 | 0.28 | 29.17 | 0.51 | 0.15 | 29.17 |
|  | 355 | 0.07 | 0.05 | 12.5 | 0.08 | 0.06 | 8.33 | 0.14 | 0.08 | 12.5 | 0.1 | 0.04 | 11.11 |
| *Rothia* | 697 | 0.02 | 0.01 | 8.33 | 0.04 | 0.02 | 20.83 | 0.06 | 0.03 | 20.83 | 0.04 | 0.01 | 16.67 |
| *Staphylococcus* | 1218 | 0.28 | 0.08 | 45.83 | 0.01 | 0.01 | 4.17 | 0 | 0 | 0 | 0.1 | 0.03 | 16.67 |
|  | 1481 | 0.09 | 0.03 | 33.33 | 0 | 0 | 0 | 0 | 0 | 0 | 0.03 | 0.01 | 11.11 |
|  | 1487 | 6.45 | 1.08 | 91.67 | 0.54 | 0.13 | 70.83 | 0.32 | 0.13 | 54.17 | 2.44 | 0.49 | 72.22 |
| *Streptococcus* | 1379 | 0.05 | 0.05 | 4.17 | 0.02 | 0.02 | 8.33 | 0.22 | 0.09 | 25 | 0.1 | 0.04 | 12.5 |
|  | 1380 | 0.33 | 0.1 | 50 | 0.13 | 0.05 | 33.33 | 0.25 | 0.11 | 37.5 | 0.24 | 0.05 | 40.28 |
|  | 1395 | 1.18 | 1.13 | 8.33 | 0.7 | 0.57 | 8.33 | 0.03 | 0.02 | 8.33 | 0.64 | 0.42 | 8.33 |
|  | 1400 | 0.11 | 0.04 | 33.33 | 0.17 | 0.06 | 45.83 | 0.35 | 0.08 | 62.5 | 0.21 | 0.04 | 47.22 |
|  | 1403 | 0.22 | 0.17 | 12.5 | 0.05 | 0.05 | 4.17 | 0.02 | 0.01 | 12.5 | 0.1 | 0.06 | 9.72 |
|  | 1414 | 0.12 | 0.12 | 4.17 | 0.24 | 0.18 | 8.33 | 0.2 | 0.17 | 8.33 | 0.19 | 0.09 | 6.94 |
|  | 1416 | 5.22 | 1.4 | 87.5 | 4.18 | 1.65 | 83.33 | 1.82 | 0.61 | 62.5 | 3.74 | 0.76 | 77.78 |
|  | 1421 | 0.03 | 0.01 | 16.67 | 0.03 | 0.01 | 12.5 | 0.01 | 0.01 | 8.33 | 0.02 | 0.01 | 12.5 |
|  | 1426 | 0.07 | 0.02 | 25 | 0.03 | 0.02 | 12.5 | 0.01 | 0.01 | 4.17 | 0.03 | 0.01 | 13.89 |
| *Sutterella* | 1179 | 0.12 | 0.12 | 4.17 | 0.08 | 0.05 | 12.5 | 0.41 | 0.35 | 8.33 | 0.2 | 0.12 | 8.33 |
| *Varibaculum* | 708 | 0 | 0 | 0 | 0.18 | 0.13 | 16.67 | 0.12 | 0.06 | 16.67 | 0.1 | 0.05 | 11.11 |
| *Veillonella* | 1573 | 0.61 | 0.44 | 25 | 0.18 | 0.12 | 20.83 | 0.81 | 0.35 | 45.83 | 0.53 | 0.19 | 30.56 |
|  | 1576 | 1.04 | 0.61 | 37.5 | 0.12 | 0.06 | 16.67 | 0.22 | 0.11 | 29.17 | 0.46 | 0.21 | 27.78 |
|  | 1578 | 0.11 | 0.1 | 16.67 | 0.01 | 0.01 | 4.17 | 0 | 0 | 0 | 0.04 | 0.03 | 6.94 |
|  | 1579 | 0.04 | 0.04 | 4.17 | 0 | 0 | 4.17 | 0.03 | 0.01 | 16.67 | 0.03 | 0.02 | 8.33 |
|  | 1588 | 1.74 | 0.7 | 54.17 | 0.65 | 0.36 | 54.17 | 0.95 | 0.48 | 41.67 | 1.11 | 0.31 | 50 |
| f_Bifidobacteriaceae | 448 | 0.01 | 0.01 | 4.17 | 0.02 | 0.01 | 8.33 | 0.02 | 0.01 | 12.5 | 0.02 | 0.01 | 8.33 |
| f_Coriobacteriaceae | 1908 | 0.02 | 0.02 | 4.17 | 0.08 | 0.04 | 12.5 | 0.13 | 0.07 | 16.67 | 0.08 | 0.03 | 11.11 |
| f_Enterobacteriaceae | 386 | 0.03 | 0.02 | 8.33 | 0.04 | 0.03 | 8.33 | 0.09 | 0.04 | 29.17 | 0.05 | 0.02 | 15.28 |
|  | 392 | 6.02 | 3.97 | 29.17 | 2.45 | 1.6 | 37.5 | 3.07 | 1.59 | 37.5 | 3.85 | 1.51 | 34.72 |
| f_Erysipelotrichaceae *Incertae_Sedis* | 1468 | 0.13 | 0.1 | 12.5 | 0.08 | 0.08 | 4.17 | 0.06 | 0.04 | 8.33 | 0.09 | 0.05 | 8.33 |
| f_Lachnospiraceae *Incertae_Sedis* | 1329 | 0.05 | 0.03 | 12.5 | 0.03 | 0.02 | 12.5 | 0.02 | 0.02 | 8.33 | 0.03 | 0.01 | 11.11 |
|  | 870 | 0.15 | 0.08 | 16.67 | 0.07 | 0.04 | 12.5 | 0.03 | 0.02 | 12.5 | 0.08 | 0.03 | 13.89 |
|  | 876 | 3.21 | 1.33 | 33.33 | 2.02 | 0.9 | 25 | 1.25 | 0.64 | 20.83 | 2.16 | 0.58 | 26.39 |
| Other OTUs (n=328) |  | 12.07 | 3.94 | 95.83 | 9.09 | 2.44 | 95.83 | 6.71 | 2.18 | 95.83 | 9.29 | 1.7 | 95.83 |

Table S3. Average concentrations of HMOs in a) maternal breast milk; b) infant faeces, indicating significant differences in concentrations between study timepoints (p<0.05)

a.

|  | **Concentrations of HMO, or HMO category in breast milk(μg/mL)** | | | | | | | | | | | | **p-value** |
| --- | --- | --- | --- | --- | --- | --- | --- | --- | --- | --- | --- | --- | --- |
|  | **2 weeks** | | | | **6 weeks** | | | | **12 weeks** | | | |  |
| **HMO** | **Min** | **Max** | **Average** | **SD** | **Min** | **Max** | **Average** | **SD** | **Min** | **Max** | **Average** | **SD** |  |
| 3FL | 79.4 | 1466.1 | 521.7 | 388.1 | 120.6 | 1959.0 | 779.7 | 482.3 | 115.3 | 2381.4 | 1002.7 | 567.8 | p<0.05 |
| 2'FL | 0.0 | 1839.0 | 1006.6 | 663.1 | 0.0 | 1704.5 | 839.9 | 567.6 | 0.0 | 1394.4 | 702.1 | 482.0 | p=0.058 |
| LNT+LNnT | 477.9 | 2330.6 | 1228.2 | 466.9 | 353.0 | 2228.1 | 1006.4 | 417.1 | 227.5 | 1820.8 | 781.3 | 333.8 | p<0.001 |
| LNFP III | 149.7 | 559.0 | 357.0 | 120.0 | 230.7 | 555.1 | 379.0 | 88.4 | 228.4 | 642.7 | 406.8 | 112.8 | p<0.05 |
| LNFP II | 69.2 | 1550.2 | 448.3 | 425.9 | 53.0 | 1514.8 | 431.0 | 377.2 | 68.4 | 1110.9 | 382.2 | 278.1 | p=0.95 |
| LNFP I | 0.0 | 1909.6 | 760.6 | 580.6 | 0.0 | 1335.5 | 446.5 | 366.6 | 0.0 | 1264.1 | 295.8 | 289.7 | p<0.05 |
| LNFP V | 0.0 | 186.2 | 49.6 | 58.0 | 0.0 | 203.8 | 53.7 | 54.5 | 0.0 | 136.3 | 48.7 | 40.9 | p=0.60 |
| LNH | 26.4 | 143.6 | 63.8 | 33.5 | 8.7 | 92.9 | 42.5 | 25.4 | 3.4 | 38.2 | 16.8 | 8.9 | p<0.001 |
| LNnH | 8.8 | 85.3 | 41.6 | 25.6 | 4.9 | 84.8 | 37.9 | 26.0 | 1.6 | 90.9 | 24.0 | 21.7 | p=0.20 |
| p-LNH | 0.0 | 50.6 | 11.6 | 11.1 | 0.0 | 44.1 | 8.6 | 9.9 | 0.0 | 47.3 | 8.2 | 11.9 | p<0.05 |
| LNDFH I | 31.7 | 1650.5 | 735.8 | 513.8 | 39.4 | 1311.1 | 603.1 | 409.9 | 29.5 | 1047.0 | 488.8 | 315.4 | p=0.19 |
| LNDFH II | 0.0 | 431.8 | 81.5 | 127.0 | 0.0 | 570.2 | 75.9 | 128.0 | 0.0 | 303.6 | 64.4 | 79.6 | p=0.63 |
| DFL | 0.0 | 262.7 | 80.6 | 73.8 | 0.0 | 201.2 | 66.1 | 53.5 | 0.0 | 155.1 | 71.5 | 54.6 | p=0.87 |
| 6'SL | 183.2 | 632.6 | 362.1 | 96.5 | 70.1 | 450.5 | 178.6 | 83.7 | 15.9 | 193.5 | 73.9 | 40.7 | p<0.001 |
| 3'SL | 99.5 | 233.2 | 174.8 | 34.3 | 87.5 | 209.2 | 162.9 | 29.0 | 94.9 | 233.7 | 164.2 | 35.6 | p<0.05 |
| LST c | 98.6 | 708.2 | 274.6 | 167.3 | 28.0 | 199.4 | 88.1 | 46.5 | 5.7 | 81.1 | 34.4 | 20.9 | p<0.001 |
| LST b | 53.9 | 435.1 | 193.5 | 84.2 | 71.7 | 489.4 | 185.4 | 97.4 | 33.0 | 306.6 | 147.0 | 66.7 | p=0.09 |
| LST a | 4.5 | 107.2 | 40.8 | 22.7 | 3.0 | 37.8 | 15.8 | 8.1 | 1.7 | 58.9 | 11.0 | 11.0 | p<0.001 |
| **Total** | 4412.8 | 8601.4 | 6432.7 | 1249.6 | 3588.1 | 7085.8 | 5401.0 | 1060.4 | 3206.5 | 6159.6 | 4723.7 | 840.3 | p<0.001 |

b.

|  | **Concentrations of HMO, or HMO category in infant faecal solution (μg/mL)** | | | | | | | | | | | | **p-value** |
| --- | --- | --- | --- | --- | --- | --- | --- | --- | --- | --- | --- | --- | --- |
|  | **2 weeks** | | | | **6 weeks** | | | | **12 weeks** | | | |  |
| **HMO** | **Min** | **Max** | **Average** | **SD** | **Min** | **Max** | **Average** | **SD** | **Min** | **Max** | **Average** | **SD** |  |
| 3FL | 0.0 | 1554.2 | 286.4 | 383.0 | 0.0 | 1795.2 | 305.1 | 476.9 | 0.0 | 2497.7 | 404.7 | 672.0 | p=0.34 |
| 2'FL | 0.0 | 953.1 | 217.2 | 316.3 | 0.0 | 1121.8 | 147.1 | 316.2 | 0.0 | 894.8 | 128.5 | 304.1 | p=0.41 |
| LNT+LNnT | 0.2 | 2015.3 | 369.4 | 536.1 | 0.0 | 909.1 | 106.0 | 215.5 | 0.0 | 606.9 | 37.8 | 123.3 | p<0.001 |
| LNFP III | 0.0 | 950.2 | 221.5 | 224.3 | 0.0 | 1245.7 | 177.2 | 278.1 | 0.0 | 785.9 | 152.1 | 250.0 | p=0.06 |
| LNFP II | 0.2 | 1468.4 | 347.1 | 436.0 | 0.0 | 1937.6 | 295.5 | 496.5 | 0.0 | 1271.4 | 169.1 | 309.8 | p<0.05 |
| LNFP I | 0.0 | 987.9 | 233.6 | 314.5 | 0.0 | 709.2 | 130.6 | 224.9 | 0.0 | 352.7 | 36.5 | 90.1 | p<0.05 |
| LNFP V | 0.0 | 127.3 | 17.7 | 33.6 | 0.0 | 36.8 | 5.6 | 11.1 | 0.0 | 35.3 | 4.4 | 10.4 | p=0.28 |
| LNH | 0.0 | 419.3 | 25.0 | 84.8 | 0.0 | 25.6 | 4.1 | 7.4 | 0.0 | 5.5 | 0.7 | 1.7 | p<0.05 |
| LNnH | 0.0 | 70.6 | 5.8 | 14.6 | 0.0 | 38.7 | 2.9 | 8.3 | 0.0 | 14.6 | 0.6 | 3.0 | p<0.05 |
| p-LNH | 0.0 | 149.8 | 9.1 | 30.4 | 0.0 | 121.4 | 6.6 | 24.6 | 0.0 | 4.3 | 0.4 | 1.0 | p=0.07 |
| LNDFH I | 0.0 | 1731.1 | 599.8 | 533.3 | 0.0 | 2263.1 | 529.2 | 688.4 | 0.0 | 1782.7 | 244.9 | 454.5 | p<0.05 |
| LNDFH II | 0.0 | 580.1 | 90.5 | 171.3 | 0.0 | 738.0 | 57.8 | 154.6 | 0.0 | 552.0 | 60.6 | 142.5 | p=0.25 |
| DFL | 0.0 | 113.5 | 31.4 | 40.1 | 0.0 | 269.0 | 30.2 | 65.4 | 0.0 | 303.8 | 29.6 | 71.4 | p=0.63 |
| 6'SL | 0.0 | 410.1 | 161.9 | 148.7 | 0.0 | 426.8 | 82.9 | 129.3 | 0.0 | 190.8 | 31.2 | 57.4 | p<0.05 |
| 3'SL | 0.0 | 204.2 | 38.6 | 55.9 | 0.0 | 179.8 | 19.7 | 50.6 | 0.0 | 258.8 | 19.2 | 60.5 | p=0.16 |
| LST c | 0.0 | 475.9 | 160.6 | 151.2 | 0.0 | 484.9 | 78.7 | 127.8 | 0.0 | 122.8 | 20.9 | 36.9 | p<0.05 |
| LST b | 0.0 | 1355.6 | 258.5 | 340.9 | 0.0 | 2536.1 | 235.0 | 552.8 | 0.0 | 1404.4 | 112.0 | 292.6 | p<0.05 |
| LST a | 0.0 | 66.8 | 14.4 | 18.8 | 0.0 | 769.6 | 35.2 | 156.6 | 0.0 | 15.5 | 1.7 | 4.2 | p=0.09 |
| **Total** | 2.6 | 5889.6 | 3088.4 | 1780.1 | 1.7 | 6728.3 | 2249.2 | 2188.5 | 0.3 | 4922.3 | 1455.1 | 2033.2 | p<0.05 |

Table S4. Differentially abundant OTUs in faecal samples of 24 infants at two, six and 12 weeks of age associated with high and low level of HMO consumption (Kruskal-Wallis; p<0.05, FDR<0.05 where indicated with*). The higher values of the average relative abundances (RA) are marked in bold.

| **HMO** | **OTU** | **Test-Statistic** | **p** | **FDR** | **High consumption group RA** | **Low consumption group RA** |
| --- | --- | --- | --- | --- | --- | --- |
| **2′FL** | *g_Bacteroides_261* | 5.60 | 0.018 | 0.223 | **0.002** | 0 |
|  | *g_Bifidobacterium_1263** | 11.16 | 0.001 | 0.036 | **0.549** | 0.198 |
|  | *g_Bifidobacterium_681** | 16.59 | 0.000 | 0.004 | **0.094** | 0 |
|  | *g_Bifidobacterium_1227* | 5.60 | 0.018 | 0.223 | **0.001** | 0 |
|  | *g_Bifidobacterium_1335* | 5.60 | 0.018 | 0.223 | **0.001** | 0 |
|  | *g_Bifidobacterium_671* | 4.36 | 0.037 | 0.247 | **0.001** | 0 |
|  | *g_Bifidobacterium_685* | 4.36 | 0.037 | 0.247 | **0.001** | 0 |
|  | *g_Bifidobacterium_1296* | 5.60 | 0.018 | 0.223 | 0 | **0.001** |
|  | *g_Clostridium_1842* | 4.36 | 0.037 | 0.247 | 0 | **0.001** |
|  | *g_Escherichia-Shigella_1210* | 4.63 | 0.031 | 0.247 | 0 | **0.001** |
|  | *g_Escherichia-Shigella_410* | 4.06 | 0.044 | 0.255 | 0.001 | **0.002** |
|  | *g_Halomonas_478* | 5.60 | 0.018 | 0.223 | **0** | 0 |
|  | *g_Streptococcus_1416* | 4.09 | 0.043 | 0.255 | 0.016 | **0.047** |
|  | *g_Veillonella_1588* | 4.84 | 0.028 | 0.247 | 0.006 | **0.022** |
|  | Other | 4.63 | 0.031 | 0.247 | 0.045 | **0.094** |
| **3FL** | *g_Bifidobacterium_1263** | 12.04 | 0.001 | 0.023 | **0.529** | 0.235 |
|  | *g_Bifidobacterium_685* | 7.81 | 0.005 | 0.124 | **0.002** | 0 |
|  | *g_Bifidobacterium_1227* | 4.94 | 0.026 | 0.208 | **0.002** | 0.001 |
|  | *g_Bifidobacterium_671* | 7.65 | 0.006 | 0.124 | **0.002** | 0.000 |
|  | *g_Bifidobacterium_1335* | 5.07 | 0.024 | 0.208 | **0.002** | 0.001 |
|  | *g_Bifidobacterium_680* | 6.41 | 0.011 | 0.165 | **0.001** | 0 |
|  | *g_Bifidobacterium_452* | 4.09 | 0.043 | 0.215 | **0.001** | 0 |
|  | *g_Bifidobacterium_1296* | 4.45 | 0.035 | 0.215 | 0 | **0.001** |
|  | *g_Enterococcus_1698* | 5.23 | 0.022 | 0.208 | **0.002** | 0 |
|  | *g_Enterococcus_1702** | 12.10 | 0.001 | 0.023 | **0.017** | 0.002 |
|  | *g_Finegoldia_1899* | 4.45 | 0.035 | 0.215 | 0 | **0.001** |
|  | *g_Lactobacillus_1528* | 5.45 | 0.020 | 0.208 | **0.018** | 0.006 |
|  | *g_Parabacteroides_284* | 4.11 | 0.043 | 0.215 | **0.003** | 0 |
|  | *g_Staphylococcus_1218* | 4.04 | 0.044 | 0.215 | 0 | **0.001** |
|  | *g_Staphylococcus_1487* | 6.41 | 0.011 | 0.165 | 0.010 | **0.034** |
|  | *g_Sutterella_1179* | 4.09 | 0.043 | 0.215 | **0.006** | 0 |
|  | *g_Varibaculum_708* | 4.09 | 0.043 | 0.215 | **0.001** | 0 |
|  | *g_Veillonella_1588* | 5.60 | 0.018 | 0.208 | 0.003 | **0.016** |
| **3′SL** | f_Lachnospiraceae *Incertae_Sedis_1329* | 4.85 | 0.028 | 0.151 | **0.001** | 0 |
|  | *g_Bifidobacterium_1263* | 4.93 | 0.026 | 0.151 | **0.418** | 0.267 |
|  | *g_Bifidobacterium_681** | 10.87 | 0.001 | 0.043 | **0.104** | 0.028 |
|  | *g_Bifidobacterium_1227* | 7.87 | 0.005 | 0.055 | **0.003** | 0 |
|  | *g_Bifidobacterium_1335* | 7.87 | 0.005 | 0.055 | **0.002** | 0 |
|  | *g_Bifidobacterium_685* | 6.33 | 0.012 | 0.084 | **0.002** | 0 |
|  | *g_Bifidobacterium_671* | 6.24 | 0.013 | 0.084 | **0.002** | 0 |
|  | *g_Bifidobacterium_680* | 6.25 | 0.012 | 0.084 | **0.001** | 0 |
|  | *g_Bifidobacterium_452* | 4.28 | 0.039 | 0.198 | 0 | **0.001** |
|  | *g_Escherichia-Shigella_422* | 8.67 | 0.003 | 0.055 | 0.021 | **0.129** |
|  | *g_Escherichia-Shigella_410* | 6.98 | 0.008 | 0.080 | 0.001 | **0.002** |
|  | *g_Escherichia-Shigella_1174* | 9.13 | 0.003 | 0.055 | 0 | **0.001** |
|  | *g_Escherichia-Shigella_1210* | 8.19 | 0.004 | 0.055 | 0 | **0.001** |
|  | *g_Escherichia-Shigella_1192** | 11.85 | 0.001 | 0.043 | 0 | **0.001** |
|  | *g_Escherichia-Shigella_382* | 5.67 | 0.017 | 0.107 | 0 | **0** |
|  | *g_Staphylococcus_1487* | 3.95 | 0.047 | 0.214 | **0.026** | 0.025 |
|  | *g_Varibaculum_708* | 4.18 | 0.041 | 0.198 | **0.001** | 0 |
|  | *g_Veillonella_1579* | 6.47 | 0.011 | 0.084 | 0 | **0.001** |
|  | *g_Veillonella_1588* | 8.10 | 0.004 | 0.055 | 0.004 | **0.022** |
| **6′SL** | *g_Bacteroides_250* | 4.20 | 0.040 | 0.440 | **0.002** | 0 |
|  | *g_Bifidobacterium_1495* | 4.26 | 0.039 | 0.440 | 0 | **0.001** |
|  | *g_Collinsella_1057* | 3.79 | 0.051 | 0.497 | **0.002** | 0.001 |
|  | *g_Haemophilus_470* | 4.81 | 0.028 | 0.410 | 0.001 | **0.007** |
|  | *g_Staphylococcus_1218* | 5.45 | 0.020 | 0.410 | 0 | **0.002** |
|  | *g_Staphylococcus_1481* | 5.16 | 0.023 | 0.410 | 0 | **0.001** |
|  | *g_Streptococcus_1379* | 5.01 | 0.025 | 0.410 | **0.002** | 0 |
|  | *g_Streptococcus_1380* | 7.72 | 0.005 | 0.410 | 0.001 | **0.003** |
|  | *g_Varibaculum_708* | 6.68 | 0.010 | 0.410 | **0.002** | 0 |
| **DFL** | f_Coriobacteriaceae*_1908* | 7.14 | 0.008 | 0.425 | 0 | **0.002** |
|  | *g_Bacteroides_233* | 3.85 | 0.050 | 0.425 | 0 | **0.001** |
|  | *g_Bacteroides_250* | 3.96 | 0.047 | 0.425 | 0.001 | **0.002** |
|  | *g_Bacteroides_266* | 4.12 | 0.042 | 0.425 | 0.001 | **0.004** |
|  | *g_Collinsella_1057* | 4.96 | 0.026 | 0.425 | 0 | **0.003** |
|  | *g_Eggerthella_600* | 4.45 | 0.035 | 0.425 | 0 | **0.002** |
|  | *g_Haemophilus_470* | 4.22 | 0.040 | 0.425 | **0.008** | 0.001 |
|  | *g_Varibaculum_708* | 5.80 | 0.016 | 0.425 | 0 | **0.001** |
|  | *g_Veillonella_1573* | 4.45 | 0.035 | 0.425 | **0.012** | 0.001 |
| **LNDFH I** | f_Enterobacteriaceae*_386* | 4.26 | 0.039 | 0.339 | **0.001** | 0 |
|  | *g_Actinomyces_707* | 4.26 | 0.039 | 0.339 | **0.001** | 0 |
|  | *g_Bifidobacterium_671* | 4.77 | 0.029 | 0.339 | **0.002** | 0.001 |
|  | *g_Bifidobacterium_685* | 4.89 | 0.027 | 0.339 | **0.002** | 0.001 |
|  | *g_Enterococcus_1702* | 10.79 | 0.001 | 0.089 | **0.015** | 0.003 |
|  | *g_Flavonifractor_1518* | 4.26 | 0.039 | 0.339 | 0 | **0.001** |
|  | *g_Parabacteroides_286* | 6.41 | 0.011 | 0.328 | 0.001 | **0.011** |
|  | *g_Streptococcus_1416* | 5.76 | 0.016 | 0.339 | 0.012 | **0.049** |
|  | *g_Streptococcus_1421* | 6.68 | 0.010 | 0.328 | 0 | **0.000** |
|  | *g_Streptococcus_1426* | 5.44 | 0.020 | 0.339 | 0 | **0.001** |
| **LNDFH II** | *g_Actinomyces_695* | 4.27 | 0.039 | 0.476 | 0 | **0.009** |
|  | *g_Bifidobacterium_1263* | 6.33 | 0.012 | 0.258 | **0.516** | 0.318 |
|  | *g_Enterococcus_1698* | 6.71 | 0.010 | 0.258 | **0.003** | 0 |
|  | *g_Enterococcus_1702* | 4.40 | 0.036 | 0.476 | **0.016** | 0.003 |
|  | *g_Halomonas_478* | 3.81 | 0.051 | 0.476 | **0** | 0 |
|  | *g_Lactobacillus_1528* | 9.53 | 0.002 | 0.088 | **0.019** | 0.005 |
|  | *g_Streptococcus_1400* | 9.66 | 0.002 | 0.088 | **0.003** | 0.001 |
| **LNFP I** | *g_Actinomyces_707* | 4.27 | 0.039 | 0.259 | **0.001** | 0 |
|  | *g_Bacteroides_233* | 4.75 | 0.029 | 0.232 | **0.001** | 0.001 |
|  | *g_Bifidobacterium_1263* | 7.02 | 0.008 | 0.117 | **0.450** | 0.221 |
|  | *g_Bifidobacterium_681** | 21.93 | 0.000 | 0.000 | **0.162** | 0.004 |
|  | *g_Bifidobacterium_1227* | 9.06 | 0.003 | 0.064 | **0.003** | 0 |
|  | *g_Bifidobacterium_1335* | 9.06 | 0.003 | 0.064 | **0.003** | 0 |
|  | *g_Bifidobacterium_685* | 6.20 | 0.013 | 0.124 | **0.002** | 0 |
|  | *g_Bifidobacterium_671* | 6.20 | 0.013 | 0.124 | **0.002** | 0 |
|  | *g_Bifidobacterium_1296* | 6.71 | 0.010 | 0.119 | 0 | **0.001** |
|  | *g_Clostridium_1792* | 4.28 | 0.039 | 0.259 | 0.002 | **0.027** |
|  | *g_Streptococcus_1400* | 8.84 | 0.003 | 0.064 | **0.003** | 0.001 |
|  | *g_Sutterella_1179* | 5.46 | 0.019 | 0.169 | **0.006** | 0 |
|  | Other | 7.60 | 0.006 | 0.101 | 0.073 | **0.120** |
| **LNFP II** | f_Enterobacteriaceae*_386* | 3.93 | 0.048 | 0.180 | **0.001** | 0.000 |
|  | f_Lachnospiraceae *Incertae_Sedis_870* | 4.12 | 0.042 | 0.174 | 0 | **0.002** |
|  | *g_Actinomyces_707* | 6.68 | 0.010 | 0.083 | **0.001** | 0 |
|  | *g_Bacteroides_233* | 5.01 | 0.025 | 0.146 | **0.001** | 0 |
|  | *g_Bacteroides_243* | 6.68 | 0.010 | 0.083 | **0.003** | 0 |
|  | *g_Bacteroides_250* | 6.68 | 0.010 | 0.083 | **0.002** | 0 |
|  | *g_Bifidobacterium_1227* | 5.16 | 0.023 | 0.143 | **0.003** | 0.002 |
|  | *g_Bifidobacterium_1263* | 9.57 | 0.002 | 0.033 | **0.546** | 0.274 |
|  | *g_Bifidobacterium_1335* | 5.16 | 0.023 | 0.143 | **0.002** | 0.001 |
|  | *g_Bifidobacterium_671* | 11.03 | 0.001 | 0.026 | **0.003** | 0 |
|  | *g_Bifidobacterium_680* | 9.30 | 0.002 | 0.033 | **0.001** | 0 |
|  | *g_Bifidobacterium_681* | 6.55 | 0.011 | 0.083 | 0.084 | **0.085** |
|  | *g_Bifidobacterium_685* | 11.21 | 0.001 | 0.026 | **0.003** | 0 |
|  | *g_Collinsella_1057* | 4.26 | 0.039 | 0.169 | **0.001** | 0 |
|  | *g_Enterococcus_1698* | 4.48 | 0.034 | 0.169 | **0.003** | 0 |
|  | *g_Enterococcus_1702* | 4.28 | 0.039 | 0.169 | **0.013** | 0.005 |
|  | *g_Haemophilus_470* | 7.18 | 0.007 | 0.083 | 0 | **0.008** |
|  | *g_Parabacteroides_284* | 4.06 | 0.044 | 0.174 | 0 | **0.002** |
|  | *g_Staphylococcus_1218* | 9.30 | 0.002 | 0.033 | 0 | **0.002** |
|  | *g_Staphylococcus_1481* | 5.44 | 0.020 | 0.142 | 0 | **0.001** |
|  | *g_Staphylococcus_1487* | 11.51 | 0.001 | 0.026 | 0.011 | **0.032** |
|  | *g_Varibaculum_708* | 4.48 | 0.034 | 0.169 | **0.002** | 0 |
|  | Other | 4.34 | 0.037 | 0.169 | 0.034 | **0.093** |
| **LST c** | *g_Actinomyces_695* | 6.68 | 0.010 | 0.170 | **0.032** | 0 |
|  | *g_Clostridium_1792* | 6.79 | 0.009 | 0.170 | **0.011** | 0.001 |
|  | *g_Collinsella_1057* | 6.68 | 0.010 | 0.170 | **0.002** | 0 |
|  | *g_Staphylococcus_1218* | 6.68 | 0.010 | 0.170 | 0 | **0.002** |
|  | *g_Streptococcus_1380* | 11.90 | 0.001 | 0.049 | 0 | **0.003** |
|  | *g_Streptococcus_1395* | 4.26 | 0.039 | 0.445 | **0.008** | 0 |
|  | *g_Streptococcus_1403* | 4.26 | 0.039 | 0.445 | 0 | **0.001** |
| **LNFP III** | *g_Actinomyces_707* | 5.44 | 0.020 | 0.124 | **0.001** | 0 |
|  | *g_Bifidobacterium_1263* | 4.97 | 0.026 | 0.150 | **0.504** | 0.313 |
|  | *g_Bifidobacterium_681** | 10.72 | 0.001 | 0.023 | **0.120** | 0.034 |
|  | *g_Bifidobacterium_685** | 12.84 | 0.000 | 0.016 | **0.003** | 0 |
|  | *g_Bifidobacterium_1227** | 9.18 | 0.002 | 0.030 | **0.003** | 0.001 |
|  | *g_Bifidobacterium_671** | 12.65 | 0.000 | 0.016 | **0.003** | 0 |
|  | *g_Bifidobacterium_1335** | 9.34 | 0.002 | 0.030 | **0.003** | 0.001 |
|  | *g_Bifidobacterium_680** | 10.69 | 0.001 | 0.023 | **0.001** | 0 |
|  | *g_Enterococcus_1702* | 5.41 | 0.020 | 0.124 | **0.015** | 0.005 |
|  | *g_Escherichia-Shigella_1192* | 5.44 | 0.020 | 0.124 | 0 | **0.001** |
|  | *g_Escherichia-Shigella_422* | 6.04 | 0.014 | 0.124 | 0.022 | **0.116** |
|  | *g_Halomonas_478* | 4.26 | 0.039 | 0.212 | **0** | 0 |
|  | *g_Parabacteroides_355* | 5.60 | 0.018 | 0.124 | 0 | **0.003** |
|  | *g_Staphylococcus_1487** | 10.07 | 0.002 | 0.026 | 0.015 | **0.028** |
|  | *g_Streptococcus_1426* | 5.75 | 0.016 | 0.124 | 0 | **0.001** |
|  | *g_Varibaculum_708* | 5.44 | 0.020 | 0.124 | **0.001** | 0 |
| **LNFP V** | f_Enterobacteriaceae_392 | 4.43 | 0.035 | 0.171 | 0.008 | **0.101** |
|  | f_Lachnospiraceae *Incertae_Sedis_876* | 7.31 | 0.007 | 0.090 | **0.034** | 0.006 |
|  | *g_Bacteroides_222* | 8.57 | 0.003 | 0.074 | **0.054** | 0.007 |
|  | *g_Bacteroides_261* | 4.53 | 0.033 | 0.170 | **0.011** | 0 |
|  | *g_Bacteroides_267* | 4.14 | 0.042 | 0.183 | **0.006** | 0 |
|  | *g_Bifidobacterium_1263** | 18.02 | 0.000 | 0.001 | **0.475** | 0.156 |
|  | *g_Bifidobacterium_1296* | 5.17 | 0.023 | 0.143 | 0 | **0.001** |
|  | *g_Clostridium_1639* | 4.53 | 0.033 | 0.170 | 0 | **0.002** |
|  | *g_Clostridium_1789* | 7.84 | 0.005 | 0.089 | 0 | **0.001** |
|  | *g_Clostridium_1792** | 18.33 | 0.000 | 0.001 | 0.001 | **0.028** |
|  | *g_Collinsella_1057* | 5.39 | 0.020 | 0.135 | **0.002** | 0 |
|  | *g_Eggerthella_600* | 4.93 | 0.026 | 0.153 | **0.002** | 0 |
|  | *g_Enterobacter_1189* | 6.16 | 0.013 | 0.104 | 0 | **0.001** |
|  | *g_Parabacteroides_284* | 7.21 | 0.007 | 0.090 | **0.003** | 0 |
|  | *g_Parabacteroides_286* | 6.61 | 0.010 | 0.098 | **0.009** | 0.001 |
|  | *g_Staphylococcus_1487* | 9.09 | 0.003 | 0.074 | 0.013 | **0.032** |
|  | *g_Streptococcus_1395* | 6.16 | 0.013 | 0.104 | 0 | **0.002** |
|  | *g_Streptococcus_1416* | 6.63 | 0.010 | 0.098 | 0.031 | **0.055** |
|  | *g_Veillonella_1573* | 4.30 | 0.038 | 0.175 | 0.002 | **0.014** |
|  | *g_Veillonella_1588* | 5.71 | 0.017 | 0.122 | 0.004 | **0.024** |
| **LNH** | f_Coriobacteriaceae*_1908* | 3.90 | 0.048 | 0.175 | **0.001** | 0 |
|  | f_Enterobacteriaceae*_392* | 4.67 | 0.031 | 0.145 | 0.025 | **0.069** |
|  | *g_Actinomyces_695* | 6.99 | 0.008 | 0.061 | 0 | **0.032** |
|  | *g_Actinomyces_704* | 5.89 | 0.015 | 0.095 | 0 | **0.003** |
|  | *g_Actinomyces_707* | 3.90 | 0.048 | 0.175 | **0** | 0 |
|  | *g_Bacteroides_222* | 3.90 | 0.048 | 0.175 | **0.055** | 0.044 |
|  | *g_Bacteroides_261* | 4.62 | 0.032 | 0.145 | **0.009** | 0 |
|  | *g_Bifidobacterium_1263** | 20.45 | 0.000 | 0.000 | **0.480** | 0.161 |
|  | *g_Bifidobacterium_681** | 21.88 | 0.000 | 0.000 | **0.116** | 0 |
|  | *g_Bifidobacterium_1227** | 12.26 | 0.000 | 0.007 | **0.003** | 0 |
|  | *g_Bifidobacterium_1335** | 12.26 | 0.000 | 0.007 | **0.003** | 0 |
|  | *g_Bifidobacterium_685** | 10.37 | 0.001 | 0.014 | **0.002** | 0 |
|  | *g_Bifidobacterium_671** | 10.37 | 0.001 | 0.014 | **0.002** | 0 |
|  | *g_Bifidobacterium_680* | 6.93 | 0.008 | 0.061 | **0.001** | 0 |
|  | *g_Bifidobacterium_1296** | 12.84 | 0.000 | 0.007 | 0 | **0.001** |
|  | *g_Clostridium_1639* | 5.16 | 0.023 | 0.134 | 0 | **0.002** |
|  | *g_Clostridium_1789* | 8.88 | 0.003 | 0.025 | 0 | **0.001** |
|  | *g_Clostridium_1792* | 9.23 | 0.002 | 0.023 | 0.002 | **0.027** |
|  | *g_Collinsella_1057* | 3.90 | 0.048 | 0.175 | **0.001** | 0 |
|  | *g_Parabacteroides_355* | 4.62 | 0.032 | 0.145 | **0.002** | 0 |
|  | *g_Staphylococcus_1487* | 4.79 | 0.029 | 0.145 | 0.021 | **0.037** |
|  | *g_Streptococcus_1395* | 6.02 | 0.014 | 0.094 | 0.000 | **0.019** |
|  | *g_Veillonella_1588* | 3.94 | 0.047 | 0.175 | 0.006 | **0.022** |
|  | Other* | 16.51 | 0.000 | 0.001 | 0.042 | **0.165** |
| **LNnH** | *g_Actinomyces_704* | 8.75 | 0.003 | 0.054 | 0 | **0.004** |
|  | *g_Bifidobacterium_1263* | 6.51 | 0.011 | 0.085 | **0.417** | 0.241 |
|  | *g_Bifidobacterium_681** | 15.41 | 0.000 | 0.008 | **0.114** | 0.005 |
|  | *g_Bifidobacterium_1227* | 6.97 | 0.008 | 0.080 | **0.003** | 0 |
|  | *g_Bifidobacterium_1335* | 7.06 | 0.008 | 0.080 | **0.002** | 0 |
|  | *g_Bifidobacterium_685* | 5.43 | 0.020 | 0.115 | **0.002** | 0 |
|  | *g_Bifidobacterium_671* | 5.52 | 0.019 | 0.115 | **0.002** | 0 |
|  | *g_Bifidobacterium_1296** | 10.65 | 0.001 | 0.026 | 0 | **0.001** |
|  | *g_Bifidobacterium_1495* | 7.84 | 0.005 | 0.069 | 0 | **0.001** |
|  | *g_Clostridium_1629* | 5.79 | 0.016 | 0.115 | 0 | **0.007** |
|  | *g_Clostridium_1789* | 5.03 | 0.025 | 0.136 | 0 | **0.001** |
|  | *g_Escherichia-Shigella_410* | 4.75 | 0.029 | 0.150 | **0.002** | 0.001 |
|  | *g_Escherichia-Shigella_422* | 7.69 | 0.006 | 0.069 | **0.070** | 0.039 |
|  | *g_Staphylococcus_1218** | 13.02 | 0.000 | 0.013 | 0 | **0.003** |
|  | *g_Staphylococcus_1481* | 6.67 | 0.010 | 0.085 | 0 | **0.001** |
|  | *g_Staphylococcus_1487** | 10.50 | 0.001 | 0.026 | 0.013 | **0.048** |
|  | *g_Streptococcus_1400* | 3.89 | 0.049 | 0.235 | **0.003** | 0.001 |
|  | Other | 5.67 | 0.017 | 0.115 | 0.057 | **0.146** |
| **LST a** | f_Bifidobacteriaceae*_448* | 6.65 | 0.010 | 0.189 | 0 | **0.000** |
|  | f_Lachnospiraceae *Incertae_Sedis_1329* | 4.88 | 0.027 | 0.215 | **0.001** | 0 |
|  | f_Lachnospiraceae *Incertae_Sedis_870* | 6.49 | 0.011 | 0.189 | **0.001** | 0 |
|  | f_Lachnospiraceae *Incertae_Sedis_876* | 6.69 | 0.010 | 0.189 | **0.037** | 0.001 |
|  | *g_Bacteroides_243* | 4.00 | 0.046 | 0.331 | 0 | **0.002** |
|  | *g_Escherichia-Shigella_1174* | 5.46 | 0.020 | 0.215 | 0 | **0.001** |
|  | *g_Escherichia-Shigella_1192* | 8.45 | 0.004 | 0.185 | 0 | **0.001** |
|  | *g_Escherichia-Shigella_1210* | 5.25 | 0.022 | 0.215 | 0 | **0.001** |
|  | *g_Escherichia-Shigella_382* | 4.91 | 0.027 | 0.215 | 0 | **0** |
|  | *g_Escherichia-Shigella_422* | 5.42 | 0.020 | 0.215 | 0.022 | **0.119** |
|  | *g_Halomonas_478* | 4.88 | 0.027 | 0.215 | **0** | 0 |
|  | *g_Veillonella_1588* | 8.17 | 0.004 | 0.185 | 0.004 | **0.018** |
| **LST b** | *g_Actinomyces_707* | 4.26 | 0.039 | 0.622 | **0.001** | 0 |
|  | *g_Bifidobacterium_1296* | 5.44 | 0.020 | 0.427 | 0 | **0.001** |
|  | *g_Bifidobacterium_681* | 5.84 | 0.016 | 0.427 | **0.149** | 0.045 |
|  | *g_Collinsella_1057* | 6.68 | 0.010 | 0.427 | **0.002** | 0 |
|  | *g_Staphylococcus_1218* | 6.47 | 0.011 | 0.427 | 0 | **0.002** |
| **pLNH** | *g_Bacteroides_261* | 4.49 | 0.034 | 0.198 | 0.009 | 0 |
|  | *g_Bifidobacterium_1263** | 15.91 | 0.000 | 0.002 | **0.457** | 0.173 |
|  | *g_Bifidobacterium_681** | 16.21 | 0.000 | 0.002 | **0.118** | 0.028 |
|  | *g_Bifidobacterium_1227** | 10.16 | 0.001 | 0.022 | **0.003** | 0 |
|  | *g_Bifidobacterium_1335** | 10.16 | 0.001 | 0.022 | **0.003** | 0 |
|  | *g_Bifidobacterium_685** | 8.40 | 0.004 | 0.041 | **0.002** | 0 |
|  | *g_Bifidobacterium_671** | 8.40 | 0.004 | 0.041 | **0.002** | 0 |
|  | *g_Bifidobacterium_680* | 5.98 | 0.015 | 0.105 | **0.001** | 0 |
|  | *g_Bifidobacterium_1296** | 15.57 | 0.000 | 0.002 | 0 | **0.002** |
|  | *g_Clostridium_1639* | 7.27 | 0.007 | 0.068 | 0 | **0.004** |
|  | *g_Clostridium_1789* | 5.36 | 0.021 | 0.128 | 0 | **0.001** |
|  | *g_Clostridium_1792* | 6.68 | 0.010 | 0.077 | 0.003 | **0.022** |
|  | *g_Eggerthella_600* | 6.76 | 0.009 | 0.077 | **0.002** | 0 |
|  | *g_Rothia_697* | 5.64 | 0.018 | 0.117 | 0 | **0.001** |
|  | *g_Staphylococcus_1487* | 3.98 | 0.046 | 0.225 | 0.020 | **0.032** |
|  | *g_Veillonella_1588* | 3.97 | 0.046 | 0.225 | 0.006 | **0.022** |
|  | Other* | 10.08 | 0.001 | 0.022 | 0.063 | **0.136** |
| **LNT and LNnT** | f_Bifidobacteriaceae*_448* | 4.26 | 0.039 | 0.154 | **0** | 0 |
|  | f_Coriobacteriaceae*_1908* | 6.68 | 0.010 | 0.065 | **0.002** | 0 |
|  | *g_Bifidobacterium_1263** | 18.97 | 0.000 | 0.001 | **0.561** | 0.162 |
|  | *g_Bifidobacterium_681* | 5.82 | 0.016 | 0.081 | 0.054 | **0.060** |
|  | *g_Bifidobacterium_1296** | 7.96 | 0.005 | 0.046 | 0 | **0.001** |
|  | *g_Bifidobacterium_1227** | 10.69 | 0.001 | 0.016 | **0.002** | 0 |
|  | *g_Bifidobacterium_1335** | 10.69 | 0.001 | 0.016 | **0.002** | 0 |
|  | *g_Bifidobacterium_452* | 6.68 | 0.010 | 0.065 | **0.001** | 0 |
|  | *g_Bifidobacterium_671* | 10.69 | 0.001 | 0.016 | **0.002** | 0 |
|  | *g_Bifidobacterium_680* | 6.68 | 0.010 | 0.065 | **0.001** | 0 |
|  | *g_Bifidobacterium_685* | 10.69 | 0.001 | 0.016 | **0.002** | 0 |
|  | *g_Clostridium_1789* | 5.44 | 0.020 | 0.081 | 0 | **0.001** |
|  | *g_Clostridium_1792** | 8.03 | 0.005 | 0.046 | 0.002 | **0.027** |
|  | *g_Eggerthella_600* | 6.02 | 0.014 | 0.081 | **0.002** | 0 |
|  | *g_Escherichia-Shigella_1174** | 9.30 | 0.002 | 0.029 | **0.001** | 0 |
|  | *g_Escherichia-Shigella_1192* | 5.44 | 0.020 | 0.081 | **0.001** | 0 |
|  | *g_Escherichia-Shigella_1210* | 5.75 | 0.016 | 0.081 | **0.001** | 0 |
|  | *g_Escherichia-Shigella_410** | 12.93 | 0.000 | 0.014 | **0.002** | 0 |
|  | *g_Escherichia-Shigella_422* | 5.77 | 0.016 | 0.081 | 0.068 | **0.072** |
|  | *g_Parabacteroides_284* | 5.44 | 0.020 | 0.081 | **0.002** | 0 |
|  | *g_Staphylococcus_1487** | 7.64 | 0.006 | 0.050 | 0.010 | **0.035** |
|  | Other | 5.72 | 0.017 | 0.081 | 0.053 | **0.153** |
| *FDR<0.05 |  |  |  |  |  |  |
